# The Role of Snake Venom Proteins in Inducing Inflammation Post-Envenomation: An Overview on Mechanistic Insights and Treatment Strategies

**DOI:** 10.3390/toxins16120519

**Published:** 2024-12-02

**Authors:** Sudharshan Rao, Nisha Reghu, Bipin Gopalakrishnan Nair, Muralidharan Vanuopadath

**Affiliations:** 1 School of Biotechnology, Amrita Vishwa Vidyapeetham, Kollam 690 525, Kerala, India; 2 Systems Biology Ireland, University College Dublin, Belfield, D04 V1W8 Dublin, Ireland

**Keywords:** snake venom, inflammation, inflammasomes, sterile inflammation, complement activation, antivenom, venom proteins

## Abstract

The intricate combination of organic and inorganic compounds found in snake venom includes proteins, peptides, lipids, carbohydrates, nucleotides, and metal ions. These components work together to immobilise and consume prey through processes such as paralysis and hypotension. Proteins, both enzymatic and non-enzymatic, form the primary components of the venom. Based on the effects they produce, venom can be classified as neurotoxic, hemotoxic, and cytotoxic. Studies have shown that, after envenomation, proteins in snake venom also contribute significantly to the induction of inflammatory responses which can either have systemic or localized consequences. This review delves into the mechanisms by which snake venom proteins trigger inflammatory responses, focusing on key families such as phospholipase A_2_, metalloproteinases, serine proteases, C-type lectins, cysteine-rich secretory proteins, and L-amino acid oxidase. In addition, the role of venom proteins in activating various inflammatory pathways, including the complement system, inflammasomes, and sterile inflammation are also summarized. The available therapeutic options are examined, with a focus on antivenom therapy and its side effects. In general, this review offers a comprehensive understanding of the inflammatory mechanisms that are triggered by snake venom proteins and the side effects of antivenom treatment. All these emphasize the need for effective strategies to mitigate these detrimental effects.

## 1. Introduction

Proteins and peptides are the major constituents of snake venom that primarily aid in prey immobilization and killing via hypotension and paralysis. In addition to proteins, snake venom is composed of organic and inorganic constituents including metal ions, lipids, carbohydrates, nucleosides, and nucleotides. Proteins belonging to non-enzymatic and enzymatic families are the major components of snake venom [1]. These proteins are mainly involved in mediating several pharmacological mechanisms in victims’ bodies following envenomation. In general, depending on the mode of action, snake venom is broadly classified as hemotoxic and neurotoxic. The venom of snake species belonging to Viperidae is rich in hemotoxins and the Elapidae family has neurotoxins in abundance [2].

The venom toxins induce both systemic and local effects in a victim through several mechanisms. For example, systemic toxins exert their biological effects by binding to specific targets. Neurotoxins and cytotoxins/cardiotoxins identified from Elapids belong to this category. Neurotoxins affect both the peripheral and central nervous system, disrupting muscle coordination that results in neuromuscular paralysis. Whereas cardiotoxins induce muscle contraction and depolarization by binding to specific receptors present in cardiac muscle cells. Similarly, hemolytic toxins present in viper venom are either involved in hemolysis, i.e., red blood cell destruction or disruption of the blood coagulation machinery. Phospholipase A_2_ (PLA_2_) present in the venom is an example of a locally acting toxin that induces inflammation, pain, and necrosis at the bite site. However, in association with other proteins, it is known to mediate systemic effects too [3,4]. In addition to PLA_2_s, several other venom proteins also have immunomodulatory effects. They may act either alone or in combination with other proteins in mediating these effects. A recent review highlights the immune mechanisms triggered by snake venom metalloproteases and C-type lectins (CTLs) associated with thromboinflammation [3]. In this regard, along with these proteins, this review describes the molecular mechanisms involved in inducing inflammation by snake venom proteins and the other factors inducing inflammation post-envenomation.

## 2. Snakebite-Induced Inflammation and the Role of Different Snake Venom Proteins

Inflammation is triggered upon the entry of antigens, in this case snake venom, into the host. The initiators of inflammatory responses are leukocytes, primarily resident macrophages and dendritic cells that recognize the antigens and phagocytose them, hence they are regarded as professional phagocytes. These cells secrete chemokines and attract neutrophils to the site of inflammation via a process known as transmigration [5], in which neutrophils begin to express cell adhesion proteins that help them to bind to the endothelium and extravasate. This process is accompanied by fluid exudation that leads to one of the inflammatory symptoms known as edema [5]. Upon extravasation, these neutrophils respond diversely to the already present inflammatory state and further induce inflammation. Some of their effects are the orchestration of coagulation, laying out neutrophil extracellular traps (NETs) [6], activating other immune cells including B cells and T cells, recruiting and activating monocytes and macrophages, and further promoting platelet production [5]. Studies have shown not just a one-way communication, but an elaborate conversation between these inflammatory mediators [7].

Other mediators of inflammation are pro- and anti-inflammatory cytokines, chemokines, anaphylatoxins, vasoactive amines (histamines and serotonins), eicosanoids (leukotrienes, thromboxanes, prostaglandins), peptides such as bradykinin, and free radicals of oxygen and nitrogen, to name a few [8]. All these molecules function in harmony to elicit the inflammatory responses triggered either by invasive compounds or compounds from within the body (sterile inflammation). Studies have shown that snake venom proteins induce these effects after envenomation and the components that are involved in inducing inflammation are described briefly below: 

### 2.1. Phospholipase A_2_

PLA_2_s are one of the most studied venom protein families not just in terms of structure and classification, but also in ways they elicit immune responses post-envenomation. They are esterolytic enzymes, involved in hydrolyzing glycerophospholipids resulting in the release of lysophosphatidic and arachidonic acid. PLA_2_s are categorized into groups I and II according to the presence of disulfide bonds. It is known that group I and II PLA_2_s are commonly found in Elapid and viper venoms, respectively [9]. Reports indicate that snake venom PLA_2_s (svPLA_2_) induce several pharmacological effects such as hemorrhage, edema, myotoxicity, neurotoxicity, cardiotoxicity, and tissue damage [10,11,12,13]. Compared to Elapid venoms, PLA_2_s are more abundant in viper species, and this substantiates the fact that tissue disruption and necrosis are predominantly associated with viper bites. In addition to the above-mentioned pharmacological mechanisms, PLA_2_s are mainly responsible for inducing inflammation after a snake bite [14].

Based on the presence of amino acids, lysine, and asparagine, at the 49th position in the protein sequence, the group II svPLA_2_s are classified as classic and variant. The former contains an asparagine [Asp49] while the latter contains a lysine [Lys49] [15]. The classic svPLA_2_s have a catalytic activity that hydrolyses the ester bond of glycerophospholipids at the SN_2_ position, which depends on Ca^2+^ ions. On the contrary, the variant svPLA_2_ has nearly no catalytic effect but has been shown to possess damaging capabilities on the membrane [12,16,17]. Though the catalytic activities vary between classic and variant svPLA_2_s, both have been shown to induce a range of inflammatory responses, that include severe local edema and leukocyte infiltration at the site of toxin entry [18,19,20].

svPLA_2_s have great tendencies to stimulate and/or recruit various immune cells either directly or indirectly. Mast cell degranulation was observed by *Bothrops jararacussu* svPLA_2_s, bothropstoxin-I (BthTX-I) and bothropstoxin-II (BthTX-II) [21], and piratoxin-I from *Bothrops pirajai* venom [22]. In vitro experiments demonstrated that piratoxin-I and bothropstoxin-I stimulated the chemotaxis of neutrophils [23] by releasing leukotriene B4 (LTB4) and platelet-activating factor (PAF) upon binding to the surface heparan of neutrophils. Neutrophil migration was activated by G-protein-coupled receptors (GPCRs) via the protein kinase C (PKC) pathway [24]. Catalytic and non-catalytic secretory PLA_2_ (sPLA_2_s), such as MT-II, MT-III, BthTX-I, BthTX-II [22], BJ-PLA_2_-I [25], BnSP-7 [26], and BatroxPLA_2_ [27], from *Bothrops* species induced polymorphonuclear and mononuclear cells’ influx at the site of envenomation [18,28].

svPLA_2_s also induce the production of various cytokines and chemokines, such as LTB4, IL-1β, IL-6, and tumor necrosis factor-alpha, TNF-α [18], that further contribute significantly to the chemotaxis of leukocytes [29,30,31]. Bbil-TX from *Bothropsis bilineata* stimulated the production of IL-6 in mice models [31], and BmatTX-I and BmatTX-II were shown to upregulate the secretion of IL-1β in mice neutrophil cell cultures [32]. Menaldo DL et al. showed that BatroxPLA_2_ induced the production of IL-6, Prostaglandin E2 (PGE2), and LTB4 by macrophage cell culture from mice [27]. BaPLA2 and BaTX-II triggered the release of TNF-α in macrophages [33]. Boene et al. [34] showed that IL-1β was secreted via nucleotide-binding domain, leucine-rich-containing family, and pyrin domain-containing-3 (NLRP3) inflammasomes in mice upon injection with a Lys49-PLA_2_, BthTX-I, that also induced inflammation by lactate dehydrogenase and cellular damage by creatine kinase release into the plasma [34,35,36]. In another study, the secretion of IL-1β was observed via NLRP3 inflammasome upon detecting the released ATP by P2X7 receptors and signaled through the caspase recruitment domain (ASC) and caspase 1/11 [37].

In addition to stimulation and recruitment of leukocytes, svPLA_2_s also play a major role in inducing the effector functions of the immune system. A study demonstrated that phagocytosis by macrophages is activated by *Bothrops* svPLA_2_s. MT-II significantly enhanced phagocytic activity through all receptor classes, and MT-III enhanced it via mannose and β-glucan receptors by assuming that there may be some molecular regions involved in this effect that are different from the catalytic sites [28]. A study conducted on BaltTX-I and BaltTX-II from *B. alternatus* showed that BaltTX-II did not induce phagocytosis by macrophages; however, BaltTX-I induced phagocytosis in macrophages through complement receptors [38]. H_2_O_2_ was released by macrophages induced by MT-II and MT-III from *Bothrops* sp., where MT-III was more potent [39]. Superoxide release by macrophages has also been demonstrated by BaltTX-I and Balt-TX-II via the PKC pathway [38]. H_2_O_2_ release has also been noted in neutrophils upon induction with BaTX-II via the PI3K pathway, in addition to the secretion of cytokines such as IL-1β, IL-8, LTB4, and neutrophil extracellular traps (NETs) [40]. So far, all sPLA_2_s have shown pro-inflammatory activities; however, a study conducted on Crotoxin B from *Crotalus durissus terrificus* has shown anti-inflammatory effects by inhibiting macrophage mobility and phagocytic capabilities [41,42].

svPLA_2_s are also involved in inducing edema through the release of prostaglandins. Intraperitoneal injections of MT-II [43] and MT-III [44,45] into mice have demonstrated the release of prostaglandin D2 (PGD2). In other similar studies, injections of Batrox-PLA_2_ [27] and BJ-PLA_2_-I [25] showed a sustained release of prostaglandin E2 (PGE2). As a result of both sets of experiments, PGD2 and PGE2 showed signs of vasodilation that led to edema [46,47]. sPLA_2_s from *B*. *asper* induced thromboxane and leukotriene B4 (LTB4) release, in vivo [18]. In addition to the release of LTB4, an Asp49 sPLA_2_ from *B. atrox* induced lipoxin and PGE2 [27]. Peritoneal leukocytes in murine models showed upregulated expression of COX-2 via NF-κB upon injection of MT-II [43] and MT-III [45]. This resulted in an increased expression of prostaglandins in macrophages of mice. To support this evidence, COX-2 and PGE2 expression by MT-II and MT-III was inhibited by TPCK, an IκB phosphorylation inhibitor [43,48]. The study also demonstrated that MT-II and MT-III phosphorylate PKC, PI3K, and PTK in macrophages [43,48]. COX-2 and PGE2 production were positively regulated, via NF-κB, because of PKC and P38MAPK stimulation by MT-III [49,50,51].

**Figure 1:**
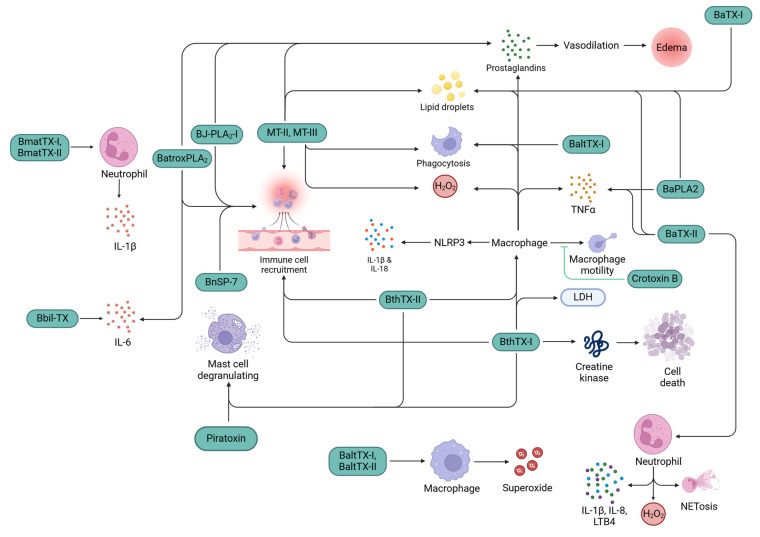
Inflammatory mechanisms induced by snake venom PLA_2_s (green color boxes) isolated from various snake species. TNF-α, tumor necrosis factor-alpha; IL, interleukin; NLRP3, nucleotide-binding domain, leucine-rich–containing family, pyrin domain–containing-3; H_2_O_2_ hydrogen peroxide; LTB4 leukotriene B4; NETosis, neutrophil extracellular traps; LDH, lactate dehydrogenase.

There has been a recent focus on understanding lipid metabolization and the consequent lipid droplet (LD) formation, which initiated an investigation as a marker for atherosclerosis and obesity [52,53,54]. In addition, LD formation is also associated with inflammation [55,56,57] and their accumulation in immunocompetent cells [58,59]. The pharmacological potential of LDs has fueled interest in exploring their interaction with snake venom in recent years. Studies have demonstrated that exposure to MT-II [49], MT-III [60], BaTX-I, BaTX-II, and BaPLA_2_ leads to an increase in LD formation within mouse macrophages [33]. MT-II and MT-III induced LD synthesis and accumulation via PI3K, PKC, p38, and ERK1/2 in murine macrophages [61]. svPLA_2_s also generate free fatty acids from membrane phospholipids. These free fatty acids bind to toll-like receptor -2 (TLR2) and signal via MyD88 adaptor molecules to release inflammatory mediators, resulting in LD accumulation in macrophages [62]. There may be more TLRs at play during this signaling event that require better investigation. CD36 receptor expression is upregulated by MT-III via PPAR-β/δ which, in turn, recognizes the free fatty acids and induces LD accumulation [63,64]. MT-III has also been shown to activate diacylglycerol acyltransferase and acyl-CoA cholesterol acyltransferase to synthesize triacylglycerol and cholesterol, respectively, in both mice macrophages [65] and in human monocytes [66], eventually leading to LD accumulation. Figure 1 shows a schematic representation of the possible mechanisms through which several snake venom PLA_2_ proteins induce inflammation.

### 2.2. Snake Venom Metalloproteinase

Snake venom metalloproteinases (SVMP) are a family of proteases with molecular masses of more than 50 kDa and are named so for their association with divalent metal ions such as zinc and cobalt to attain maximum activity. These proteins are categorized into four classes based on their functional domains: P-I SVMPs containing just the metalloproteinase domain; P-II SVMPs containing metalloproteinase and disintegrin domains, but also found with just the disintegrin domain; P-III SVMPs contain cysteine-rich domains in addition to disintegrins and metalloproteinase domains; and P-IV SVMPs contain all the P-III domains and a lectin-like domain [67].

Before we move ahead, it is a prerequisite to understand that metalloproteases (MPs) and matrix metalloproteinases (MMPs) are different but interact via key events such as inflammation [68,69]. Edema, degranulation of mast cells, and leukocyte infiltration are the most commonly attributed effects of SVMPs [70,71,72]. It has also been observed that SVMPs cause hemorrhage by loosening the connective tissue of blood vessels [73]. A very similar set of observations was reported by McKay [74] and Ownby [75] through the experiments conducted to study the effects of snake venoms on capillaries. Another set of studies showed the hydrolysis of laminin, nidogen, entactin, type IV collagen, fibronectin, and proteoglycans [73] in addition to various other targets of SVMPs [76]. SVMPs have been shown to play a role in coagulopathies [77]; however, their involvement in hemorrhage is a complex relationship with MMPs. Capillary basement proteins and ECM are targeted by SVMPs to disrupt the hemostasis and cause capillary rupture [78,79,80]. A P-III SVMP from *Crotalus simus* enzymatically cleaved the basement membrane and, thereby, led to pulmonary hemorrhage and edema [81]. CsH1, an SVMP isolated from *C. simus*, induced pulmonary hemorrhage activity, basement membrane components were hydrolyzed, induced inflammation in the lungs, and hemorrhage mediated through inflammatory mediators [81]. Similarly, another P-III SVMP, bothropasin, from *B. jararaca* venom, was responsible for edema, hemorrhage, and necrosis [82]. Atroxlysin-I, a P-I class of SVMPs from *B. atrox*, possessed hemorrhagic and fibrinogenolytic activity. It also has the capability to cleave the ECM and inhibit platelet function [83]. Damage to endothelial cell integrity was reported in a study conducted on salmosin, a disintegrin isolated from *Agkistrodon halys brevicaudus* venom [84]. These cellular damages would lead to the generation of DAMPs which would then result in a positive feedback loop of cytokine signaling, potentially leading to a cytokine storm [85].

**Figure 2:**
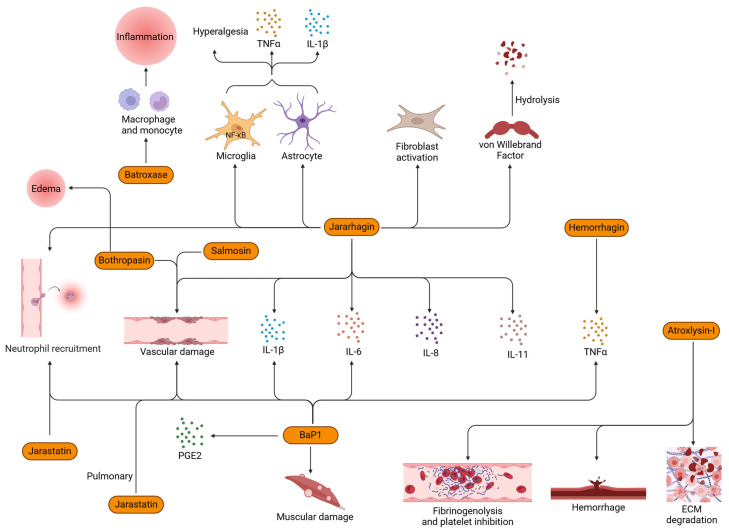
Inflammatory mechanisms induced by SVMPs (orange color boxes) isolated from various snake species. TNF-α, tumor necrosis factor-alpha; IL, interleukin; NF-κB, nuclear factor kappa-light-chain-enhancer of activated B cells; PGE2, prostaglandin E2; vWF, von Willebrand factor.

As seen with other venom protein families, SVMPs promote proinflammatory cytokine release. One of the most widely studied SVMPs, jararhagin, isolated from *Bothrops jararaca*, induced the release of inflammatory mediators such as IL-1β, IL-6, IL-8, and IL-11 and damaged vascular tissue [86,87]. An in vivo study on jararhagin also induced the production and release of IL-1β and TNF-α and activated glial cells in the spinal cord via the NF-κB pathway, resulting in the first-ever observation of the involvement of spinal cord glial cells and astrocytes in venom-induced mechanical hyperalgesia [88]. In addition to jararhagin, hemorrhagin isolated from *Echis pyramidum leakeyi* venom activated TNF-α in mice models [89]. BaP1 from *B. asper* venom induced the production of IL-1β, IL-6, and MMPs in vivo [90]; joint hypernociception through TNF-α and PGE2 [91]; neutrophil recruitment and cytokine release [92]; and local tissue damage in muscular and endothelial tissues [93]. Clissa et al. showed that the SVMP jararhagin stimulated macrophages to mediate the release of inflammatory cytokines in addition to the recruitment of inflammatory cells, without directly influencing the chemotactic activity [94]. In addition, jararhagin is also known to activate fibroblasts [87,95] and hydrolyze von Willebrand factor (vWF) [96]. Jarastatin, a disintegrin isolated from B. jararaca venom, proved to be a strong chemoattractant of human neutrophils in vitro, signaling via PI3K and MAPK pathways [97]. A P-III SVMP promoted inflammation through the recruitment of neutrophils into the parenchyma of the lungs [81]. The upregulation of chemokines such as CXCL1 and CXCL2 by jararhagin may also explain the increased recruitment of neutrophils [87]. Batroxase, from *B. atrox,* promoted acute inflammation in vitro and in vivo through macrophages and mast cells [27]. Figure 2 shows a schematic representation of the possible mechanisms through which several SVMP proteins induce inflammation.

### 2.3. Snake Venom Serine Protease

Most abundantly found in viperid venoms, SVSPs are monomeric glycoproteins weighing about 26–76 kDa and are categorized under the proteolytic class of enzymes [98]. Their direct effects on the coagulation machinery make them a potent coagulator by inducing platelet aggregation and activation of coagulation factors [99,100]. SVSPs were also able to induce the production of cytokines and chemokines in vitro and in vivo. KnBa, from *Bitis arietans*, increased THP1 macrophage cell viability at least by 90%, upregulating the production of IL-1β, TNF, and IL-6 [101]. KnBa also upregulated chemokines such as IL-8, RANTES, MCP-1, and IP-1; however, KnBa was not involved in PGE2 production [101]. The functions of SVSPs were also varied in different species of *Bothrops*. BpirSP27 and BpirSP41 are two SVSPs isolated from *Bothrops pirjai* that did not elicit the basic signs of inflammation—edema, pain, and leukocyte recruitment [102]. *Bothrops alternus* and *Bothrops moojeni* venom demonstrated an increase in the levels of pain and edema in mouse models [103]. Cdtsp2, an SVSP purified from *Crotalus durissus terrificus*, capable of degrading fibrinogen, acts on a GPCR leading to inflammation, thrombosis, and disruption of homeostasis [104]. Their effector functions are signaled via protein kinase C (PKC) and phospholipase C (PLC), generating inositol triphosphate (IP3) and diacylglycerol (DAG). In the same study, the authors observed that Cdtsp2 acted on protease-activated receptors, PAR1 and PAR2 on the mast cells to induce edema with suspected production of PGE2. They were also observed to act on PAR3 and PAR4 which are expressed on platelets to alter their activity.

### 2.4. C-Type Lectin

CTLs in snake venom are known as snaclecs and belong to the non-enzymatic class of snake venom proteins. In other organisms, including humans, they exist as homodimers and bind calcium and sugar residues. Whereas the CTLs found in venom proteins lack the typical calcium/sugar-binding loop and have adapted to interact with a broad spectrum of biologically significant proteins and receptors. They are mostly heterodimeric with active α and β subunits covalently linked by disulfide linkages [105]. Snaclecs from snake venom interact with various receptors and proteins, including C-type lectin-like receptor 2 (CLEC-2); coagulation factors; vWF, GPIb, and GPVI receptors on platelets; and α2β1 receptors of integrins. Hence, they are known to have platelet aggregation inhibitory activities [105]. In viper venom, hemostasis and inflammation are closely linked, with thromboinflammation being well-documented in these species [3]. The involvement of CLEC-2 receptors in inducing thromboinflammation is also explored in detail [106]. The report indicated that aggretin, a CTL from *Calloselasma rhodostoma*, binds to the CLEC-2 receptors present on monocytes and macrophages, resulting in the production and release of pro-inflammatory cytokines such as IL-6 and TNF-α through JNK and ERK phosphorylation (MAPKs) [106]. In another study, aggretin also possessed pro-angiogenic activities by inducing the expression of VEGF, through the PI3K, Akt, and Erk1/2 pathways upon integrin α2β1 activation [107]. In addition to aggretin, agglucetin, isolated from *Agkistrodon acutus*, also possessed pro-angiogenic activities through the FAK, PI3K, and Akt pathways [108].

**Figure 3:**
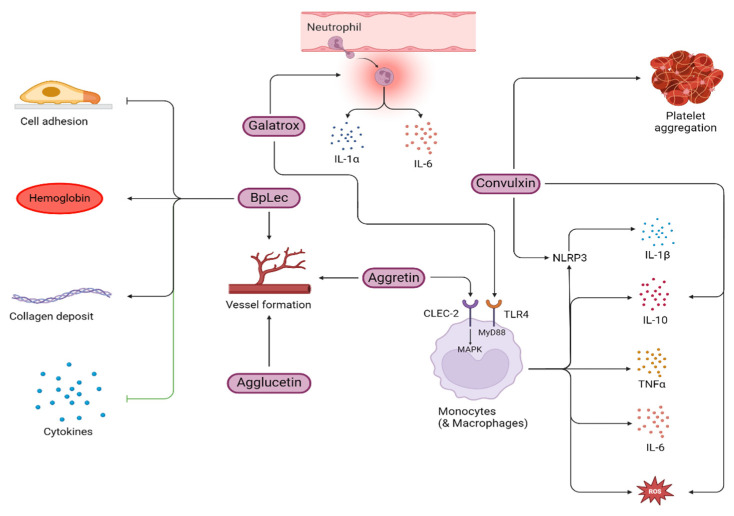
Inflammatory mechanisms induced by snake venom CTLs (purple color boxes) isolated from various snake species. TNF-α, tumor necrosis factor-alpha; IL, interleukin; ROS, reactive oxygen species; NLRP3, nucleotide-binding domain, leucine-rich–containing family, pyrin domain–containing-3; TLR-4, toll-like receptor-4.

A C-type lectin-like protein known as convulxin (CVX), extracted from the venom of the *Crotalus durissus* snake is known to stimulate platelet aggregation but was found to be non-toxic to peripheral blood mononuclear cells and also did not affect cell growth or IL-2 release. However, it induced IL-10 secretion and ROS production via monocytes and activated the NLRP3 inflammasome leading to IL-1β secretion. This effect is mediated through its interaction with the Dectin-2 receptor (a CTL receptor) highlighting the role of CVX in modulating immune cell functions and inflammation [109]. CTLs can modulate immune responses through various mechanisms. For instance, Galatrox, a glycan-binding protein from *Bothrops atrox* snake venom promotes neutrophil migration and induces the release of pro-inflammatory cytokines, such as IL-1α and IL-6 both in vitro and in vivo. Additionally, it also stimulates macrophages to produce pro-inflammatory mediators through the TLR4-MyD88 signaling pathway suggesting its role in mediating the toxicity of *B. atrox* venom [110]. A study on BpLec, from *Bothrops pauloensis* demonstrated increased hemoglobin levels and blood vessel formation in vivo. In addition, BpLec also inhibited cell adhesion and pro-inflammatory cytokines [111]. BpLec possessed the properties of an angiogenic and inflammatory modulator. However, the authors surprisingly noted an increase in neutrophil count though the proinflammatory cytokine levels were diminished. Figure 3 shows a schematic representation of the possible mechanisms through which several snake venom CTL proteins induce inflammation.

### 2.5. Cysteine-Rich Secretory Protein

As part of the CAP [cysteine-rich secretory proteins (CRISPS), antigen 5 (Ag5), and pathogenesis-related 1 (Pr-1)] protein superfamily, CRISPs are the most intriguing molecules to snake venom biologists. They are non-enzymatic proteins having a molecular mass of 20–30 kDa with all 16 cysteine residues strictly conserved [112]. They are widely seen in diverse organisms including various snake families such as the Elapids, Viperids, and the Colubrids and mediate a wide variety of biological functions [113,114]. However, their intricate biological mechanisms are still far from being understood, but many hypotheses have been developed to explain their roles in eliciting inflammation, edema, necrosis, cell death, and other pharmacologically important outcomes. CRISPs are not directly involved in causing death in mammals but contribute to various effects that can severely disrupt the homeostasis in the host body. The ion channel blocking, myotoxic, and proinflammatory activities of snake venom CRISPs have been explored in detail by several groups [112,115,116,117].

A study conducted on a CRISP (Bj-CRP) isolated from *B. jararaca* revealed that it induced inflammation by promoting neutrophil recruitment and IL-6 production [118]. In line with this, another study demonstrated that a CRISP (BaltCRP) isolated from *Bothrops alternatus* induced the expression of IL-1β and IL-10, in addition to IL-6 [119].

**Figure 4:**
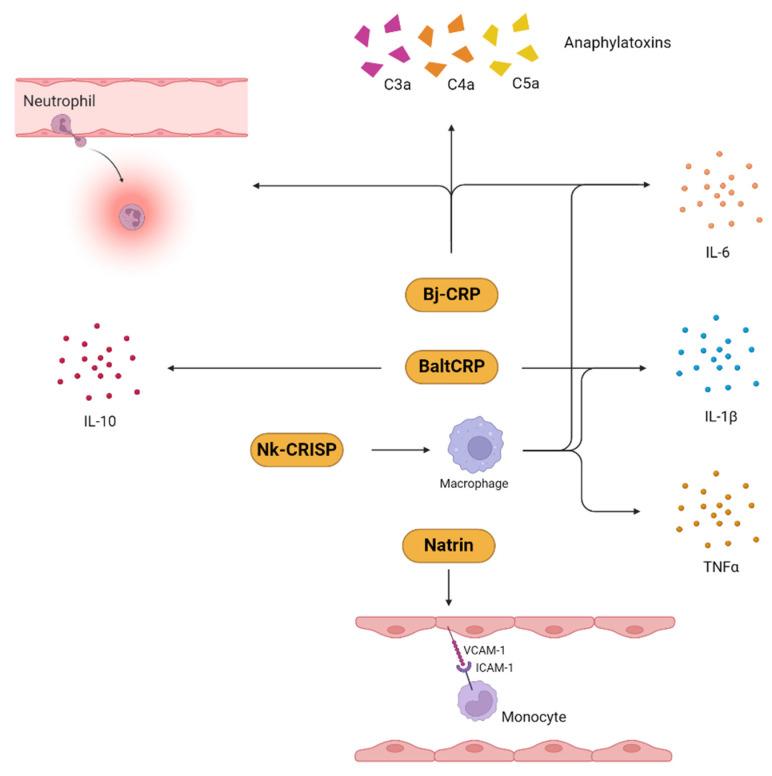
Inflammatory mechanisms induced by snake venom CRISPs (squash color boxes) isolated from various snake species. TNF-α, tumor necrosis factor-alpha; IL, interleukin; ICAM-1, intercellular adhesion molecule-1; VCAM-1, vascular cell adhesion molecule-1; C3a, complement component 3a; C4a, complement component 4a; C5a, complement component 5a.

The CRISP natrin, from *Naja atra*, mediated its inflammatory activity by inducing the expression of monocytic cell adhesion molecules such as the vascular cell adhesion molecule (VCAM-1), the intracellular adhesion molecule (ICAM-1), and E-selectin [120]. The production of proinflammatory molecules, such as IL-6, TNF-α, and IL-1β, by Nk-CRISP, a CRISP from *Naja kaouthia*, was suggested to be through the involvement of the toll-like receptors TLR-2 and TLR-4 [121]. Figure 4 shows a schematic representation of the possible mechanisms through which several snake venom CRISP proteins induce inflammation.

### 2.6. L-Amino Acid Oxidase

Like other components of snake venom, L-amino acid oxidase (svLAAO) is also involved in inducing inflammatory responses such as edema, hemolysis, and myotoxicity [122,123]. Although the specific roles of svLAAO are not clearly understood, crude venom from the *Bothrops* species has been shown to induce necrosis in dermal cells, also referred to as dermonecrosis [124,125]. In addition to this, studies have also reported apoptotic activity of svLAAO through the generation of H_2_O_2_ [126]. H_2_O_2_ is known to trigger inflammation and blood clots, and may also have necrotic and hemorrhagic effects. Therefore, the severity of envenomation would also be influenced by the amount of H_2_O_2_ generated by LAAOs. This has been supported by other studies where sequestration of H_2_O_2_ decreased the apoptotic activity of svLAAO [122,127]. Other studies have demonstrated that edema is induced in mice models when treated with svLAAO isolated from *Cerastes cerastes* [128,129,130] and *Bungarus fasciatus* [131]. LAAO from *Bungarus* also induced severe myotoxicity, accumulation of inflammatory cells, and myolysis [131]. The role of different snake venom proteins in inducing inflammation post envenomation are summarized in Table 1.

## 3. Inflammasomes: Role in Snake Venom-Induced Inflammation

Inflammasomes can be activated under sterile and non-sterile conditions of inflammation. Any component of the venom that causes cytotoxicity or cellular disruption, can lead to the activation of inflammasome [34,37,101,109,137,138,139,140]. Reports suggest that snake venom PLA_2_s, CTLs, SVSPs, and LAAOs are involved in the activation of inflammasomes resulting in inflammatory responses [34,37,101,109,137,138,139,140]. Inflammasomes mediate the inflammatory responses through caspase-1-dependent mechanisms resulting in the production of proinflammatory cytokines IL-1β and IL-18 and inducing pyroptosis [141].

sPLA_2_s of Viperidae venoms share structural and functional similarities with secretory PLA_2_s and are one of the most potent activators of inflammasomes [142,143]. Studies conducted on *Bothrops* sp. PLA_2_s have shown that they have myotoxic activity and also trigger the release of IL-1 and IL-6, which leads to the speculation of the involvement of inflammasomes [18,39,144]. A study on BthTX-I, a Lys49-sPLA_2_ from *B. jararacussu*, induced IL-1β release in mice muscles, but not through P2X7 receptors (which signal for inflammasome activation and assembly) [34]. On the contrary, another study conducted on BthTX-I, obtained from the same species, reported the involvement of NLRP3 and caspase activation through P2X7 receptors, mediated by ATP [37]. In an in vitro study on macrophages, both BthTX-I and BthTX-II induced the release of IL-1β and IL-18 via ASC, NLRP3, and caspase-1 [137]. Studies involving BthTX-I and II have demonstrated that these proteins activate the NLRP3 inflammasome complex contributing to inflammation post envenomation [34,139].

In a similar study conducted on sPLA_2_s from *B. moojeni*, the authors demonstrated an indirect trigger of inflammation by the release of ATP by somatosensorial neurons, which in turn activated P2X2 and P2X3 receptors [145]. Since ATP is a DAMP, the involvement of NLRP3 inflammasomes is highly likely, thus resulting in the surge of caspase-1, IL-1β, and IL-18 levels [34,146,147]. Similarly, convulxin, a CTL from *Crotalus durissus terrificus*, proved to be a potent activator of NLRP3 inflammasome in human PMBCs, in vitro [109]. Convulxin is also known to activate NF-κB and release IL-2, all contributing to the inflammatory responses [148,149]. Another CTL, BjcuL, isolated from *B. jararacussu*, activated NLRP3 inflammasomes through TLR4, in vitro. BjcuL also demonstrated the activation of NF-κB in the process, resulting in the release of IL-1β [140]. The first ROS-dependent NLRP3 activation was reported by a study conducted on Cr-LAAO isolated from *C. rhodostoma* [138]. The NLRP3 expression was noted in human neutrophils in vitro and inhibition studies were carried out to confirm the roles of ROS, NLRP3, and caspase-1 when incubated with Cr-LAAO. The involvement of *Bitis arietans* venom and Kn-Ba, an SVSP from the same species has suggested the involvement of inflammasomes in the release of IL-1β in the THP-1 cell supernatant [101]. All these studies demonstrated the proinflammatory activities of different snake venom components through inflammasome activation. However, a study has shown that caspase-1 inflammasome activation protects mouse models from the toxic effects of bee and snake venom. Caspase-1 might be inducing these protective mechanisms through membrane repair by recruiting neutrophils thereby aiding tissue repair mechanisms. The same study also suggests that inflammasomes protected the animals through the detoxification of venom components indicating the beneficial role of inflammasomes rather than their harmful effects [150]. Nevertheless, these mechanisms need to be explored in detail.

**Figure 5:**
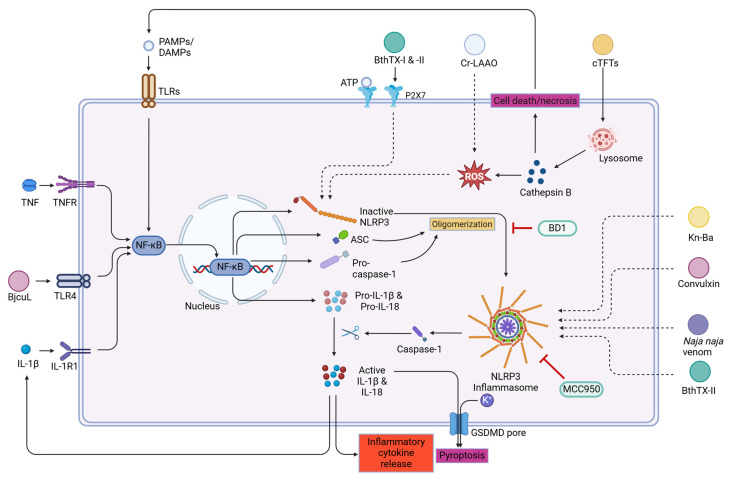
NLRP3 inflammasome activation by various snake venom proteins and its effector functions. TNF-α, tumor necrosis factor-α; IL, interleukin; NLRP3, nucleotide-binding domain, leucine-rich–containing family, pyrin domain–containing-3; ROS, reactive oxygen species; GSDMD, gasdermin D; BD1, dimethyl ester of bilirubin; ATP, adenosine triphosphate; ASC, apoptosis-associated speck-like protein; NF-κB, nuclear factor kappa-light-chain-enhancer of activated B cells; TLR-4, toll-like receptor-4; TNFR, tumor necrosis factor receptor; IL-1R1, interleukin-1 receptor 1; PAMPs/DAMPs, pathogen/damage-associated molecular patterns. The venom proteins/crude venom responsible for inducing inflammasome activation are highlighted in different colors; green- phospholipase A_2_; dark yellow-snake venom serine protease; purple-C-type lectins, white-L-amino acid oxidase; Orange-three finger toxins.

Studies performed on the venoms from the ‘big four’ snake species in India have demonstrated that only *Naja naja* venom activated the inflammasome pathways in mouse models. The findings also demonstrated that treatment using MCC950, a selective NLRP3 inflammasome inhibitor, reduced the production of IL-1β through the activation of caspase-1 pathways in mouse macrophages [151]. In another study, the same group has shown that dimethyl ester of bilirubin (BD1) inhibited the activation of MAPKs and NLRP3 inflammasomes and reduced *Naja naja* venom-induced lung toxicity [152]. All these results are suggestive of the fact that compounds inhibiting inflammasome activation might be useful in reducing inflammation and local tissue damage post-envenomation. Figure 5 shows a schematic representation of the possible mechanisms through which several snake venom proteins activate inflammasome-mediated inflammation.

## 4. Complement Pathway Activation Post Envenomation

Consisting of more than 40 interacting plasma and surface proteins, the complement system functions in sync with other immune defense systems to mediate inflammation and the clearance of antigens [153]. Its initiation can occur via one of three major pathways—classical, alternative, and lectin pathways, that all converge at the formation of C3 convertase [154,155]. This C3 convertase cleaves C3 into C3a (anaphylatoxin) and C3b (opsonin). Downstream pathways of C3 convertase activation are the generation of C5 convertases that cleave C5 into C5a and C5b. Anaphylatoxins, such as C3a, C4a, and C5a, mediate local innate immune responses such as vasodilation via mast cells, leukocyte recruitment, the release of reactive oxidative species (ROS), and cytokine-release [155]. An increase in the C3 and C5 cleavage activity has been noted during snake bite envenomation [156]. This activity may be due to the direct or indirect effect of the snake venom itself, which aggravates the inflammatory responses seen after envenomation. A study in Brazil conducted on 19 species of snake species demonstrated a significant increase in the complement components in the serum, predominantly through the classical pathway [157]. Among the numerous components of snake venom, proteomic analysis showed that the major contributors were SVMPs and SVSPs [157,158].

Numerous studies have been conducted on *Bothrops* species to understand their influence on the complement system. C-SVMP, a P-I class SVMP isolated from *B. pirajai* possessed the ability to cleave C3, C4, and C5, which increased the levels of anaphylatoxins [158]. This study also showed that this toxin was able to activate all three pathways. C-SVMPs were able to generate C3a, C5a, and SC5b-9 [159]. Other similar studies suggested the involvement of BpirLAAO-I, BjussuSP-I [160], BpirSP27, and BpirSP41 [102] in complement activation and the resulting inflammation from *B. pirajai* and *B. jararacussu*. In addition to complement cleavage, C-SVMP also induced the secretion of a chemoattractant, CXCL9/MIG, in the blood, in addition to increasing the expression of CD11b, C3aR, CD14, C5aR1, TLR2, and TLR4 in leukocytes [159]. *B. lanceolatus* triggered an increased expression of C4a and C5a, but not C3a, which was similarly observed in another study on *B. brazili* venom [157,161]. However, in the latter study, various other *Bothrops* species significantly induced the production of all the anaphylatoxins—C3a, C4a, and C5a. The authors suggest that there may be the presence of peptidases in these two Bothrops venoms that degrade only C3a. Similarly, a study conducted on *Naja annulifera* demonstrated that the C5a-C5aR1 axis is elicited by SVMPs, that induce the expression of prostaglandins, leukotrienes, and thromboxanes [162], along with the increased expression of CXCL1.

The proteolytic activities of the coagulation and complement systems inevitably lead to curiosity about the role of SVSPs since both contain serine proteases. Yamamoto et al. [163] demonstrated the C3-cleaving activity of Flavoxobin from *Trimeresurus flavoviridis* venom. This showed that an SVSP could act as a C3 convertase. In a study conducted on *Bothrops pirajai*, serine proteases BpirSP41 inhibited CP and LP hemolytic activity, while BpirSP41 and BpirSP27 inhibited AP hemolytic activity [102]. Certain snake venoms have shown complement-inhibitory activities, which we suggest may be an evolutionary adaptation of snakes to increase their venom potency. A study on *N. atra* demonstrated the ability of a class-III SVMP, atrase B, to cleave C6, C7, C8, and Factor B which concluded their anti-hemolytic properties [164]. Similarly, rFII, a recombinant fibrinogenase enzyme, sourced from *Agkistrodon acutus* cleaved C5, C6, and C9 components [165,166]. Activation of the complement system was observed by *Micrurus* spp. which significantly increased the production of anaphylatoxins such as C3a, C4a, and C5a, for which, the proposed mediators were SVMPs and SVSPs [167].

## 5. Snakebite Envenomation Induced Sterile Inflammation

Several signaling cascades are activated when inflammatory cells are drawn to the site of tissue or cell damage. These inflammatory pathways are activated through alarm signals known as damage/danger-associated molecular patterns (DAMPs) [7]. The DAMPs are composed of motifs that are highly conserved [168]. Cellular integrity might be hampered due to several physical, chemical, and environmental factors resulting in a condition known as sterile inflammation. Several reports indicate that snakebite envenomation also results in inducing sterile inflammation in the host [7,14,85]. In reaction to these, even in the absence of infections, inflammatory cells may be drawn to the site of cell injury.

**Figure 6:**
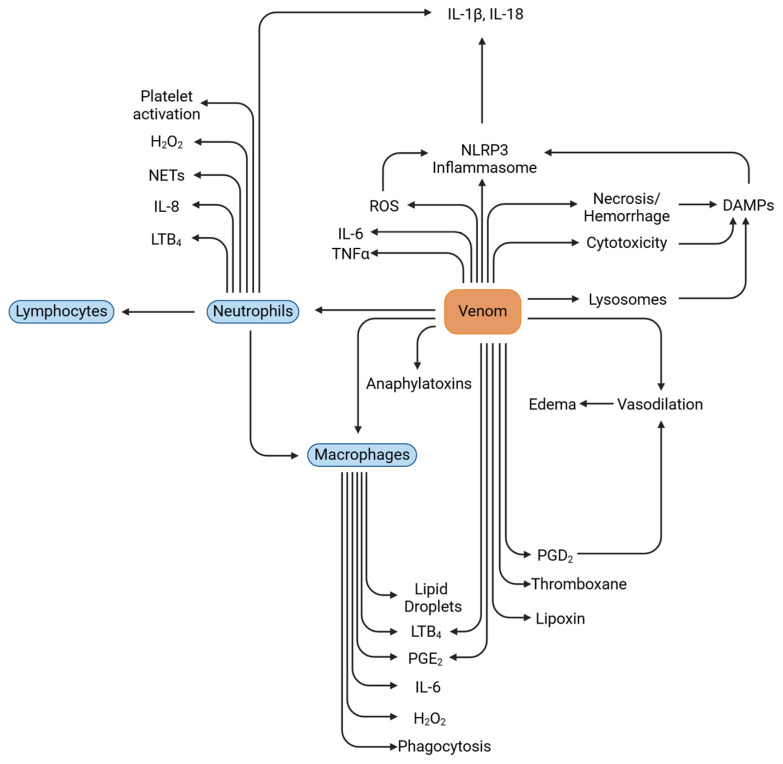
Possible mechanisms of sterile inflammation elicited by snake venom proteins post envenomation. TNF-α, tumor necrosis factor-alpha; IL, interleukin; NLRP3, nucleotide-binding domain, leucine-rich–containing family, pyrin domain–containing-3; ROS, reactive oxygen species; PGE2, prostaglandin E2; LTB4 leukotriene B4; NETs, neutrophil extracellular traps; H_2_O_2_ hydrogen peroxide; DAMPs, damage-associated molecular patterns; PGD2, prostaglandin D2.

The process termed sterile inflammation is mediated by several molecules including HMGB1, HSPs, S100B, S100A8, S100A9, MyD88 [62], and IL-6 [169,170]. One member of the HMG protein family, HMGB1, is mostly found in the cell nucleus and is essential for controlling the expression of genes. However, upon its extracellular release, HMGB1 has been observed to elicit an inflammatory response through the activation of the NF-κB pathway. HMGB1 binds to several receptors, including TLR4, TLR2, and TLR9, and the receptor for advanced glycation end products (RAGE), triggering their activation. The primary function of the S100 proteins, a class of calcium-binding proteins, is to control the build-up of calcium inside cells. HSPs (heat shock proteins) typically serve as chaperone proteins, aiding in biosynthetic processes. However, when HSPs are released into the extracellular environment due to cellular necrosis, they can trigger inflammation by activating receptors like TLR2, TLR4, and CD91. In a study conducted to check the levels of sterile inflammatory markers during snakebite, it was observed that there was a significant increase in markers like HMGB1, IL-6, HSP, and S100B [170]. The authors of the same study also showed that titanium-dioxide nanoparticles (Ti-NP) reduced the levels of these sterile inflammatory markers. They observed that Ti-NP provided greater protection against viper-venom-induced mice models than from cobra venom, which was speculated to be because of the higher levels of PLA_2_ in viper venom than in cobra venom. Apart from a very few reports mentioned above, it must be noted that the mediators of sterile inflammation post-envenomation have not been studied in detail. Figure 6 shows a schematic representation of the possible mechanisms through which several snake venom proteins induce sterile inflammation.

## 6. Snakebite Treatment

A person bitten by a venomous snake would need to seek medical attention before the serious effects of the venom start to occur. This poses a concern for healthcare systems in two ways: treatments for envenomation must be available at every healthcare facility and the effectiveness of the treatment option itself [171].

### 6.1. Antivenom

Snake antivenoms are the primary treatment for snakebite envenomation, offering lifesaving protection against the toxicity of snake venom. Large animals, such as horses or sheep, are immunized with snake venom to produce immunoglobulins, which can then be processed further using proteolytic enzymes to produce Fab or F (ab’)2 antibodies [172]. They can be polyvalent, which can neutralize several snake venoms, or monovalent, which is effective against just one kind of snake venom. Antivenom antibodies recognize and bind to venom components circulating in the blood or tissue compartments following a parenteral entry in the envenomed patients, aiding their neutralization. [173]. In India, the polyvalent antivenom (PAV) that is developed against the venom of “The Big Four”—the spectacled cobra, common krait, Russell’s viper, and saw-scaled viper—is utilized [174]. Although the PAV produced is used as a mainstay treatment against snakebite envenoming, they are effective at neutralizing systemic toxins only, and their ability to neutralize local effects is limited, leading to complications from envenomation [175].

Traditional antivenoms have several limitations due to their heterologous nature and production methods. The presence of non-human proteins in antivenoms, including the host-animal antibodies, can cause immunogenic reactions in snakebite victims, such as serum sickness or anaphylaxis. Additionally, antivenom products often experience batch-to-batch variability and may contain low or imbalanced levels of therapeutically relevant antibodies. While para-specificity can occur, antivenoms are typically most effective against the venoms of the snake species they were specifically designed for. The manufacturing process is labor intensive and low throughput, which raises concerns about the inclusion of animal-derived impurities, the risk of disease transmission, and the overall cost. Since venoms are complex mixtures of various toxins with differing toxicity, abundance, and immunogenicity, not all medically important toxins trigger a strong immune response, limiting the antivenom’s ability to neutralize all toxins [176]. Several reports have demonstrated and highlighted the necessity for developing antivenoms that are region-specific to improve the treatment outcomes in snakebite envenomed victims [175,177,178,179,180]. To streamline necessary treatment strategies, a detailed characterization of snake venom components needs to be conducted through proteomics approaches [181].

Effective treatment of envenomation requires the timely administration of antivenom, which is influenced by factors such as the antivenom dose, the amount of venom injected, and the recognition of venom proteins by the antivenom. The antivenom is administered intravenously, but the initial dose must be tailored based on the patient’s response. If signs of severe envenomation are observed, such as worsening neurotoxic effects, cardiovascular issues, or persistent incoagulable blood after 6 h, the dosage is adjusted upwards. However, without the specific snake species responsible for the bite or the precise initial dose needed, administering a high amount of antivenom poses a risk of serious adverse reactions. The challenge of determining the correct dosage is compounded by the lack of comprehensive clinical trials. Consequently, clinicians often rely on the manufacturer’s estimates, which are typically based on the antivenom’s efficacy in neutralizing venom in laboratory rodents [4]. Research has shown that not all venom proteins are recognized and bound to by antivenoms, and low molecular weight, less immunogenic proteins, even when highly lethal, do not provoke a robust immune response in the host animals which makes the antivenom less effective [179,182].

### 6.2. Treatment of the Inflammatory Symptoms After Snakebite

One of the biggest challenges posed by antivenoms is their ability to neutralize only the free-circulating venom in the blood and not against the venom that has already begun to act on its target components. Once the symptoms of an envenomation have been initiated, there is a subsequent cascade of pro-inflammatory activities leading to tissue/organ damage, and subsequently, death [175]. Due to the physical impossibility of a victim to gain instantaneous access to aid, it is important for healthcare centers to treat the symptoms of the envenomation in addition to antivenom therapy [171,175].

Antivenoms are usually supplemented with anti-inflammatory drugs such as NSAIDs, antihistamines, anticholinesterases, etc. [183] in order to reduce the body’s allergic reactions against the antivenom. Depending on the overall effect of the envenomation, specific medications may be prescribed, for example, coagulants for bites from Russell’s viper. An extensive clinical study conducted by Mahmood et al., on hospitalization cases showed significant diversity in the complications led by snake venom [184]. Prophylactics such as hydrocortisone and antihistamines are generally given to patients before antivenom injections but were found to be ineffective in many studies, probably due to the time it takes to act on the body [185,186]. In addition, antihistamines act only on already released histamine and do not confer protection against further release. However, a study that supplemented hydrocortisone with chlorphenamine reduced the antivenom reactions [187]. Depending on the severity of the antivenom reactions, either antihistamines or adrenaline is administered [188]. In addition, in the case of local tissue necrosis, the victim is also administered with tetanus toxoid as an injection [189].

## 7. Antivenom-Mediated Hypersensitivity Reactions

Antivenom administration must be cautiously approached due to the potential for multiple adverse reactions [190]. Severe reactions can occur within an hour after antivenom administration, necessitating close patient observation and continuous monitoring of vital signs to detect any adverse effects promptly. The safety of antivenom is influenced by several production parameters, such as snake venom composition, immunoglobulin composition, immunoglobulin fragment purification, and the presence of other components including preservatives. At first, antivenoms included fragment crystallizable (Fc) and fragment antigen-binding (Fab) sections of entire immunoglobulin G (IgG), which led to a number of adverse reactions. Antivenoms made of Fab or F (ab’’)2 with Fc fragments eliminated were created to lessen the adverse consequences [191]. However, subsequent research revealed that the purity and protein content of the antivenom were more critical determinants of adverse reactions than Fc-mediated complement activation. Well-purified whole IgG antivenom demonstrated comparable potency and safety to F (ab’’)2 antivenom [191]. Depending on the purification process utilized, the geographical area, and the precise type of antivenom used, the incidence rate of adverse reactions to antivenom varies [187,192,193].

Adverse hypersensitivity reactions to snake antivenom may occur as both acute (anaphylactic and pyrogenic reactions) and delayed (serum sickness) [194]. Anaphylactic reactions appear within 10–180 min after administering the antivenom. They encompass various symptoms such as itching, hives, dry cough, fever, nausea, vomiting, abdominal pain, rapid heartbeat, and diarrhea. In more severe cases, some patients may experience anaphylactic reactions characterized by low blood pressure, swelling of the skin or mucous membranes, and constriction of the airways [195,196,197]. It is possible to further categorize anaphylactic reactions as either IgE-mediated or non-IgE-mediated. Adverse events induced by early IgE antibodies are seldom documented during the treatment of antivenom. These reactions manifest in individuals who have been previously exposed to animal immunoglobulin, which generates IgE antibodies. These IgE antibodies bind to mast cells and basophils, triggering cell degranulation upon exposure to antivenom [198]. This release of chemicals like leukotrienes and prostaglandins causes vasodilation, increased permeability, muscle contractions, and increased gland function [199]. Most early reactions brought on by antivenom are not IgE-mediated. These reactions occur de novo in patients with no prior record of antivenom administration. The WHO does not recommend using an intradermal hypersensitivity test, which is common for allergic reactions, for non-IgE mediated anaphylactic reactions. Two mechanisms are proposed to explain the non-IgE mediated anaphylactic reactions: antivenom anticomplementary activity (ACA) and the presence of heterophilic antibodies [200].

Pyrogenic reactions usually develop 1–2 h after starting ASV therapy [201]. These are caused by the presence of pyrogen contaminants during the manufacturing process. These reactions can manifest as chills, rigors, fever, myalgia, headache, tachycardia, and hypotension due to blood vessel dilation. Pyrogens that are most found in antivenoms are bacterial lipopolysaccharides. Typically, these reactions occur within the first hour of antivenom infusion. Treatment involves physically cooling the patient and administering antipyretics like paracetamol to manage such reactions. In severe cases accompanied by hypotension, intravenous fluids, and adrenaline may be necessary. To prevent these reactions, strict adherence to good manufacturing practices is essential to avoid microbial contamination in the antivenom, including pyrogens. Delayed reactions, known as serum sickness, belong to type III hypersensitivity in the Gell–Coombs classification. The reaction typically occurs between 5 and 20 days after administering antivenom, and it is triggered by soluble antigen-antibody complex formation. Upon antivenom administration, the patient’s immune system produces antibodies that attach to the antivenom, resulting in the formation of antigen-antibody complexes. These immune complexes thereby can trigger the complement system and cause immune cells, particularly leukocytes, to infiltrate affected areas [202].

## 8. Prophylactic Medications for Antivenom Mediated Complications

Both pharmacologic and non-pharmacologic therapies are used to treat antivenom-induced anaphylactic responses. Non-pharmacologic interventions include airway management, fluid resuscitation and momentarily pausing the infusion of antivenom injection [190]. Potential treatments for minimizing the occurrence and intensity of antivenom reactions include the administration of corticosteroids, adrenaline, and antihistamines [183,203]. Pharmacokinetic studies have demonstrated that intramuscular delivery of epinephrine is more efficacious than subcutaneous administration making it a primary pharmacologic intervention. Patients who do not respond to intramuscular adrenaline and fluid resuscitation may require intravenous administration of adrenaline [190]. Following successful control of the reactions and achieving hemodynamic stability, the antivenom infusion may be resumed slowly. However, this can cause acute reactions to recur, thereby demanding a repeated administration of adrenaline.

Adrenaline rapidly counteracts the effects of hypersensitivity observed in anaphylaxis. It targets the cardiovascular system but can also lead to cardiac arrhythmias. Antihistamine drugs are commonly administered along with adrenaline to prevent a recurrence of anaphylaxis. These drugs are considered relatively safe. Corticosteroids are also used, as they can suppress the immune system. However, their effects may take several hours (4–6 h) to become noticeable after administration [183]. A study by Premawardhena et al. found reduced acute reactions in the serum when adrenaline was administered subcutaneously immediately prior to antivenom treatment [192]. If pyrogenic reactions occur, antipyretics (paracetamol) and physical cooling are both used as treatments for fever. In severe hypotension cases, intravenous fluids and adrenaline may be needed. Following excellent manufacturing practices will prevent microbial products from contaminating the antivenom, preventing adverse responses.

## 9. Recent Advancements

The field of snakebite envenomation has witnessed significant advancements in recent years, driven by the application of modern research techniques and a renewed focus on improving patient outcomes. The need for a better understanding of snake venoms from a multi-omics perspective is paramount to enhance the efficiency of antivenoms and to minimize their side effects [204,205]. Advances in venomics have enhanced the understanding of venom diversity, while snake venom extracellular vesicles (svEVs) may play a part in the envenomation process, though further research is necessary. Characterization of blood plasma proteome post-envenomation holds significant potential for identifying venom biomarkers, but its complexity makes direct proteomic analysis challenging. However, isolating extracellular vesicles has shown promise, offering insights into tracking venom effects and assessing antivenom effectiveness, providing a comprehensive overview of snakebite and treatment responses [206]. A study investigating *B. atrox* venom was conducted to assess the changes in systemic pathological and inflammatory responses in a mouse model. This research employed hematologic, lipidomic, and shotgun proteomic analyses to provide insights into the venom’s effects on the body’s systems [207]. A recent advancement in understanding the pathophysiology of snakebites and enhancing clinical management involves the analysis of the proteomic composition of blister fluids from envenomation patients. This highlights proteomics as a valuable diagnostic tool, capable of detecting various tissue, plasma, and inflammatory proteins resulting from the tissue-damaging effects of snake venom. It offers important insights into the pathological and inflammatory processes occurring in venom-affected tissues [208]. Proteomic analysis of exudates from tissues affected by snake venoms has emerged as an effective method for understanding the distinct patterns of tissue damage caused by crude venoms and purified toxins, such as hemorrhagic SVMPs and myotoxic phospholipase A2. This approach can also help identify differences in the effects of venoms that exhibit varying pathophysiological profiles [209]. A detailed investigation of the plasma proteome of mice treated with crude *Bothrops* venom has shown that it is involved in inducing a cascade of inflammatory responses including thromboinflammation [210,211]. Similarly, recent studies have explored innovative therapeutic approaches for snakebite envenomation, focusing on improving patient outcomes. One such approach involves the use of mesenchymal stromal cells (MSCs) to address muscle damage caused by snake venom. Early results show that the MSC secretome significantly mitigates muscle damage caused by snake venom. This research seeks to harness the regenerative potential of MSCs to promote skeletal muscle regeneration, capitalizing on their established roles in immune modulation and angiogenesis promotion [212].

Despite extensive research, our knowledge of the functional aspect of most animal toxins is still unclear. This is especially true for the processes of toxin production, storage, and the specialized anatomical structures within venom-producing tissues that might affect venom composition [213]. A highly promising tool, mass spectrometry imaging (MSI), aims to offer insights into the spatial expression of proteins by integrating protein data acquisition through mass spectrometry with visualization software [214]. MSI has been previously employed to study the spatial differentiation of snake venom glands [215,216]. A study has reported the use of matrix-assisted laser desorption/ionization mass spectrometry imaging (MALDI-MSI) combined with proteo-transcriptomic analyses to map the spatial distribution of toxins within the venom gland of the Egyptian cobra (*Naja haje*). The research uncovered significant spatial heterogeneity in toxin classes at the proteoform level, distributed across different regions of the venom gland [213]. However, a detailed investigation is warranted to evaluate the pathophysiological mechanism induced by crude venoms from a clinical perspective.

## 10. Conclusions

The intricate and varied characteristics of snake venom proteins and their functions in triggering inflammation following envenomation are emphasized to wrap up this review. Although many snake venom proteins were formerly classified according to their cytotoxic, hemotoxic, or neurotoxic properties, it is now clear that these proteins also play important roles in inducing inflammatory responses. Different clinical consequences may arise from these inflammatory effects, which might appear both locally, at the bite site, and systemically, throughout the victim’s body. Snake venom proteins can cause inflammation through a variety of mechanisms, including complement system activation, the development of inflammasome-mediated inflammation, and through sterile inflammation. These pathways may aggravate long-term recovery and elevate the risk of subsequent infections and other consequences, in addition to contributing to the initial pain, swelling, and tissue damage seen after envenomation. Therefore, it is essential to comprehend these mechanisms to create tailored treatment plans.

The effectiveness of current therapeutics methods, which mostly involve the administration of antivenom, in completely alleviating the inflammatory aftermath of snakebites is limited. Antivenoms are often made to counteract the toxins that cause potentially fatal hemotoxic or neurotoxic effects, although they might not fully treat the venom’s inflammatory components. Adjunctive treatments that can precisely block inflammatory pathways triggered by venom components are, therefore, desperately needed. Subsequent investigations must concentrate on identifying and characterizing venom proteins accountable for these inflammatory reactions. Novel therapeutic treatments that either directly block these proteins or modify the host’s inflammatory response may be developed by figuring out their precise mechanisms of action and interactions with host immune components. Furthermore, investigating the possibility of anti-inflammatory medications in conjunction with conventional antivenoms as part of a combination therapy may provide a more thorough course of treatment, enhancing the prognosis for snakebite victims. So, a better comprehension of the inflammatory pathways set off by snake venom proteins enhances our understanding of the biology of the respective venom and may create new opportunities for therapeutic intervention. These discoveries have the potential to improve patient outcomes and quality of life by lowering the burden of inflammation brought on by snakebite.

## Figures and Tables

**Table 1:** Snake venom proteins and their role in inducing inflammation.

Protein Name	Snake Venom Protein Family	Snake Species	Inflammatory Mechanisms Involved	Model Used	Reference
BthTX-I and BthTX-II	PLA_2_s	*B. jararacussu*	Leukocyte recruitment	Neutrophils from healthy human blood	[23]
			Inflammasome activation	Mice	[34]
			Mast cell degranulation	Mice	[21,22]
Piratoxin-I		*B. pirajai*	Mast cell degranulation	Mice	
MT-II and MT-III		*Bothrops* sp.	Leukocyte recruitment	Mice	[22]
		*B. asper*	Increased macrophage phagocytic activity	Mice	[28]
		*B. asper*	H_2_O_2_ release by macrophages	Mice	[39]
		*B. asper*	Activation of NF-kB	Mice	[43,45]
		*B. asper*	Vasodilation	Mice	[43,44]
BJ-PLA2		*B. jararaca*	Leukocyte recruitment	Mice	[25]
		*B. jararaca*	Vasodilation and edema	Mice	
BnSP-7		*Bothrops pauloensis*	Leukocyte recruitment	Mice	[26]
Batrox-PLA2		*B. atrox*	Leukocyte recruitment	Mice and in vitro using cells obtained from these treated mice	[27]
		*B. atrox*	Cytokine and chemokine release	Mice and in vitro using cells obtained from these treated mice	[27]
BaPLA2		*B. atrox*	TNF-α release in macrophages	In vitro, J77A.1 macrophage cell line	[33]
Bbil-TX		*Bothriopsis bilineata*	IL-6 production	Mice	[132]
BmatTX-I and BmatTX-II		*Bothrops mattogrossensis*	IL-1β production	Mice and in vitro using ELISA	[32]
BaltTX-I and BaltTX-II		*B. alternatus*	H_2_O_2_ release	Mice	[38]
BaTX-I and BaTX-II		*B. atrox*	H_2_O_2_, IL-1β, IL-8, LTB4, NETs release	Neutrophils from healthy human blood	[40]
Crotoxin B		*C. durissus terrificus*	Inhibition of macrophage phagocytic activity and motility	Mice	[41,42]
Bj-CRP	CRISPs	*B. jararaca*	Inflammation via neutrophil recruitment and IL-6 production	Mice	[118]
		*B. jararaca*	Anaphylatoxin production	In vitro, in human serum	
Balt-CRP		*B. alternatus*	Cytokine release	Mice	[119]
Natrin		*N. atra*	Promotes expression of cell adhesion molecules	In vitro, endothelial cells from the human umbilical cord and U937 monocytes	[120]
Nk-CRISP		*N. kaouthia*	Cytokine release	In vitro, THP-1	[121]
Jararhagin	SVMPs	*B. jararaca*	Cytokine release	Mice	[94,133]
		*-*	Leukocyte recruitment	Mice	
Jarastatin			Chemoattractant	In vitro, neutrophils	[97]
CsH1		*C. simus*	Pulmonary hemorrhage	Mice	[81]
Bothropasin		*B. jararaca*	Edema, hemorrhage, necrosis	In vitro	[82]
Atroxlysin-I		*B. atrox*	Hemorrhage and fibrinogenolytic	In vitro and in mice	[83]
Salmosin		*A. halys brevicaudus*	Endothelial cell disruption	In vitro, bovine capillary endothelial cell line	[84]
Hemorrhagin		*E. pyramidum leakeyi*	TNF-α production	In vitro, WEHI 164 subclone 13	[89]
BaP1		*B. asper*	Cytokine and MMP production, neutrophil recruitment, local tissue damage	In vitro assay	[90,91,92,93]
Batroxase		*B. atrox*	Inflammation through macrophages and mast cells	Mice	[27]
VaaSP-VX	SVSPs	*Vipera ammodytes*	Cleaves Factor V and factor X	In vitro assays	[134]
Kn-Ba		*B. arietans*	Cytokine and chemokine release	In vitro, THP-1 macrophages	[101,135]
		*B. arietans*	Fibrinogenolytic	In vitro assay	
		*B. arietans*	Kinin-release	In vitro assay	
Ancrod		*C. rhodostoma*	Fibrinogenolysis	In vitro assay	[136]
Batroxobin		*B. atrox*		In vitro assay	
RVV-V		*D. russelii*			
BpirSP27 and BpirSP41		*B. pirajai*	Complement activation	In vitro assay	[102]
Cdtsp2		*C. durissus terrificus*	Fibrinogenolysis	In vitro assay	[104]
		*C. durissus terrificus*	Edema	Mice	
Aggretin	CTLs	*C. rhodostoma*	VEGF induction	In vitro, HUVECs (Human Umbilical Vein Endothelial Cells)	[107]
Agglucetin		*A. acutus*	Pro-angiogenic	In vitro, HUVECs	[108]
Convulxin		*C. durissus terifficus*	Promotes platelet aggregation	In vitro assay	[109]
Galatrox		*B. atrox*	Neutrophil migration, cytokine release, stimulates macrophages	Mice and in vitro assays	[110]
BpLec		*B. pauloensis*	Pro-angiogenesis; increased hemoglobin production; inhibition of cell adhesion, cytokines, and collagen deposit	Mice	[111]

## Data Availability

No new data were created or analyzed in this study. Data sharing is not applicable to this article.

## References

[1] Jimenez-Porras J.M. (1968). Pharmacology of peptides and proteins in snake venoms. Annu. Rev. Pharmacol..

[2] Tasoulis T., Isbister G.K. (2017). A Review and Database of Snake Venom Proteomes. Toxins.

[3] Teixeira C., Fernandes C.M., Leiguez E., Chudzinski-Tavassi A.M. (2019). Inflammation Induced by Platelet-Activating Viperid Snake Venoms: Perspectives on Thromboinflammation. Front. Immunol..

[4] Gutierrez J.M., Calvete J.J., Habib A.G., Harrison R.A., Williams D.J., Warrell D.A. (2017). Snakebite envenoming. Nat. Rev. Dis. Primers.

[5] Sherwood E.R., Toliver-Kinsky T. (2004). Mechanisms of the inflammatory response. Best Pract. Res. Clin. Anaesthesiol..

[6] Perdomo J., Leung H.H.L., Ahmadi Z., Yan F., Chong J.J.H., Passam F.H., Chong B.H. (2019). Neutrophil activation and NETosis are the major drivers of thrombosis in heparin-induced thrombocytopenia. Nat. Commun..

[7] Zuliani J.P. (2023). Alarmins and inflammatory aspects related to snakebite envenomation. Toxicon.

[8] Medzhitov R. (2008). Origin and physiological roles of inflammation. Nature.

[9] Ohno M., Chijiwa T., Oda-Ueda N., Ogawa T., Hattori S. (2003). Molecular evolution of myotoxic phospholipases A_2_ from snake venom. Toxicon.

[10] Karabuva S., Brizic I., Latinovic Z., Leonardi A., Krizaj I., Luksic B. (2016). Cardiotoxic effects of the *Vipera ammodytes* ammodytes venom fractions in the isolated perfused rat heart. Toxicon.

[11] Terra A.L., Moreira-Dill L.S., Simoes-Silva R., Monteiro J.R., Cavalcante W.L., Gallacci M., Barros N.B., Nicolete R., Teles C.B., Medeiros P.S. (2015). Biological characterization of the Amazon coral *Micrurus spixii* snake venom: Isolation of a new neurotoxic phospholipase A_2_. Toxicon.

[12] Gutierrez J.M., Lomonte B. (2013). Phospholipases A2, unveiling the secrets of a functionally versatile group of snake venom toxins. Toxicon.

[13] Kamiguti A.S., Laing G.D., Lowe G.M., Zuzel M., Warrell D.A., Theakston R.D. (1994). Biological properties of the venom of the Papuan black snake (*Pseudechis papuanus*): Presence of a phospholipase A_2_ platelet inhibitor. Toxicon.

[14] Luo P., Ji Y., Liu X., Zhang W., Cheng R., Zhang S., Qian X., Huang C. (2023). Affected inflammation-related signaling pathways in snake envenomation: A recent insight. Toxicon.

[15] Krizaj I., Bieber A.L., Ritonja A., Gubensek F. (1991). The primary structure of ammodytin L, a myotoxic phospholipase A_2_ homologue from *Vipera ammodytes* venom. Eur. J. Biochem..

[16] Diaz C., Gutierrez J.M., Lomonte B., Gene J.A. (1991). The effect of myotoxins isolated from *Bothrops* snake venoms on multilamellar liposomes: Relationship to phospholipase A2, anticoagulant and myotoxic activities. Biochim. Biophys. Acta.

[17] Lomonte B., Gutierrez J.M. (2011). Phospholipases A_2_ from viperidae snake venoms: How do they induce skeletal muscle damage?. Acta Chim. Slov..

[18] Zuliani J.P., Fernandes C.M., Zamuner S.R., Gutierrez J.M., Teixeira C.F. (2005). Inflammatory events induced by Lys-49 and Asp-49 phospholipases A_2_ isolated from *Bothrops asper* snake venom: Role of catalytic activity. Toxicon. Off. J. Int. Soc. Toxinol..

[19] Teixeira C.F., Landucci E.C., Antunes E., Chacur M., Cury Y. (2003). Inflammatory effects of snake venom myotoxic phospholipases A2. Toxicon.

[20] Landucci E.C., de Castro R.C., Toyama M., Giglio J.R., Marangoni S., De Nucci G., Antunes E. (2000). Inflammatory oedema induced by the lys-49 phospholipase A_2_ homologue piratoxin-i in the rat and rabbit. Effect of polyanions and p-bromophenacyl bromide. Biochem. Pharmacol..

[21] Landucci E.C., Castro R.C., Pereira M.F., Cintra A.C., Giglio J.R., Marangoni S., Oliveira B., Cirino G., Antunes E., De Nucci G. (1998). Mast cell degranulation induced by two phospholipase A_2_ homologues: Dissociation between enzymatic and biological activities. Eur. J. Pharmacol..

[22] de Castro R.C., Landucci E.C., Toyama M.H., Giglio J.R., Marangoni S., De Nucci G., Antunes E. (2000). Leucocyte recruitment induced by type II phospholipases A_2_ into the rat pleural cavity. Toxicon.

[23] Gambero A., Landucci E.C., Toyama M.H., Marangoni S., Giglio J.R., Nader H.B., Dietrich C.P., De Nucci G., Antunes E. (2002). Human neutrophil migration in vitro induced by secretory phospholipases A_2_, a role for cell surface glycosaminoglycans. Biochem. Pharmacol..

[24] Gambero A., Thomazzi S.M., Cintra A.C., Landucci E.C., De Nucci G., Antunes E. (2004). Signalling pathways regulating human neutrophil migration induced by secretory phospholipases A2. Toxicon.

[25] Cedro R.C.A., Menaldo D.L., Costa T.R., Zoccal K.F., Sartim M.A., Santos-Filho N.A., Faccioli L.H., Sampaio S.V. (2018). Cytotoxic and inflammatory potential of a phospholipase A_2_ from *Bothrops jararaca* snake venom. J. Venom. Anim. Toxins Incl. Trop. Dis..

[26] Oliveira Cde F., Lopes Dda S., Mendes M.M., Homsi-Brandeburgo M.I., Hamaguchi A., de Alcantara T.M., Clissa P.B., Rodrigues Vde M. (2009). Insights of local tissue damage and regeneration induced by BnSP-7, a myotoxin isolated from *Bothrops* (*neuwiedi*) *pauloensis* snake venom. Toxicon.

[27] Menaldo D.L., Bernardes C.P., Zoccal K.F., Jacob-Ferreira A.L., Costa T.R., Del Lama M.P., Naal R.M., Frantz F.G., Faccioli L.H., Sampaio S.V. (2017). Immune cells and mediators involved in the inflammatory responses induced by a P-I metalloprotease and a phospholipase A_2_ from *Bothrops atrox* venom. Mol. Immunol..

[28] Zuliani J.P., Gutierrez J.M., Teixeira C. (2018). Signaling pathways involved in zymosan phagocytosis induced by two secreted phospholipases A_2_ isolated from *Bothrops asper* snake venom in macrophages. Int. J. Biol. Macromol..

[29] Arend W.P., Gabay C. (2004). Cytokines in the rheumatic diseases. Rheum. Dis. Clin. N. Am..

[30] David B.A., Kubes P. (2019). Exploring the complex role of chemokines and chemoattractants in vivo on leukocyte dynamics. Immunol. Rev..

[31] de Oliveira S., Rosowski E.E., Huttenlocher A. (2016). Neutrophil migration in infection and wound repair: Going forward in reverse. Nat. Rev. Immunol..

[32] de Moura A.A., Kayano A.M., Oliveira G.A., Setubal S.S., Ribeiro J.G., Barros N.B., Nicolete R., Moura L.A., Fuly A.L., Nomizo A. (2014). Purification and biochemical characterization of three myotoxins from *Bothrops mattogrossensis* snake venom with toxicity against Leishmania and tumor cells. BioMed Res. Int..

[33] Furtado J.L., Oliveira G.A., Pontes A.S., Setubal Sda S., Xavier C.V., Lacouth-Silva F., Lima B.F., Zaqueo K.D., Kayano A.M., Calderon L.A. (2014). Activation of J77A.1 macrophages by three phospholipases A_2_ isolated from *Bothrops atrox* snake venom. BioMed Res. Int..

[34] Boeno C.N., Paloschi M.V., Lopes J.A., Pires W.L., Setubal S.D.S., Evangelista J.R., Soares A.M., Zuliani J.P. (2019). Inflammasome Activation Induced by a Snake Venom Lys49-Phospholipase A_2_ Homologue. Toxins.

[35] Melo P.A., Homsi-Brandeburgo M.I., Giglio J.R., Suarez-Kurtz G. (1993). Antagonism of the myotoxic effects of Bothrops jararacussu venom and bothropstoxin by polyanions. Toxicon.

[36] Veronese E.L., Esmeraldino L.E., Trombone A.P., Santana A.E., Bechara G.H., Kettelhut I., Cintra A.C., Giglio J.R., Sampaio S.V. (2005). Inhibition of the myotoxic activity of *Bothrops jararacussu* venom and its two major myotoxins, BthTX-I and BthTX-II, by the aqueous extract of *Tabernaemontana catharinensis* A. DC. (Apocynaceae). Phytomed. Int. J. Phytother. Phytopharm..

[37] Raneia E.S.P.A., de Lima D.S., Mesquita Luiz J.P., Camara N.O.S., Alves-Filho J.C.F., Pontillo A., Bortoluci K.R., Faquim-Mauro E.L. (2021). Inflammatory effect of Bothropstoxin-I from *Bothrops jararacussu* venom mediated by NLRP3 inflammasome involves ATP and P2X7 receptor. Clin. Sci..

[38] Setubal S.S., Pontes A.S., Furtado J.L., Xavier C.V., Silva F.L., Kayano A.M., Izidoro L.F., Soares A.M., Calderon L.A., Stabeli R.G. (2013). Action of two phospholipases A_2_ purified from *Bothrops alternatus* snake venom on macrophages. Biochem. Biokhimiia.

[39] Zuliani J.P., Gutierrez J.M., Casais e Silva L.L., Coccuzzo Sampaio S., Lomonte B., Pereira Teixeira Cde F. (2005). Activation of cellular functions in macrophages by venom secretory Asp-49 and Lys-49 phospholipases A_2_. Toxicon.

[40] Setubal S.D.S., Pontes A.S., Nery N.M., Rego C.M.A., Santana H.M., de Lima A.M., Boeno C.N., Paloschi M.V., Soares A.M., Zuliani J.P. (2020). Human neutrophils functionality under effect of an Asp49 phospholipase A_2_ isolated from *Bothrops atrox* venom. Toxicon X.

[41] Bon C., Choumet V., Delot E., Faure G., Robbe-Vincent A., Saliou B. (1994). Different evolution of phospholipase A_2_ neurotoxins (beta-neurotoxins) from Elapidae and Viperidae snakes. Ann. N. Y. Acad. Sci..

[42] Rossetto O., Morbiato L., Caccin P., Rigoni M., Montecucco C. (2006). Presynaptic enzymatic neurotoxins. J. Neurochem..

[43] Moreira V., de Castro Souto P.C., Ramirez Vinolo M.A., Lomonte B., Maria Gutierrez J., Curi R., Teixeira C. (2013). A catalytically-inactive snake venom Lys49 phospholipase A_2_ homolog induces expression of cyclooxygenase-2 and production of prostaglandins through selected signaling pathways in macrophages. Eur. J. Pharmacol..

[44] Moreira V., Gutierrez J.M., Soares A.M., Zamuner S.R., Purgatto E., Teixeira Cde F. (2008). Secretory phospholipases A_2_ isolated from *Bothrops asper* and from *Crotalus durissus terrificus* snake venoms induce distinct mechanisms for biosynthesis of prostaglandins E_2_ and D_2_ and expression of cyclooxygenases. Toxicon.

[45] Moreira V., Gutierrez J.M., Amaral R.B., Zamuner S.R., Teixeira Cde F. (2009). Effects of *Bothrops asper* snake venom on the expression of cyclooxygenases and production of prostaglandins by peritoneal leukocytes in vivo, and by isolated neutrophils and macrophages in vitro. Prostaglandins Leukot. Essent. Fat. Acids.

[46] Kida T., Sawada K., Kobayashi K., Hori M., Ozaki H., Murata T. (2014). Diverse effects of prostaglandin E_2_ on vascular contractility. Heart Vessel..

[47] Gerritsen M.E. (1996). Physiological and pathophysiological roles of eicosanoids in the microcirculation. Cardiovasc. Res..

[48] Moreira V., Lomonte B., Vinolo M.A., Curi R., Gutierrez J.M., Teixeira C. (2014). An Asp49 phospholipase A_2_ from snake venom induces cyclooxygenase-2 expression and prostaglandin E2 production via activation of NF-kappaB, p38MAPK, and PKC in macrophages. Mediat. Inflamm..

[49] Giannotti K.C., Leiguez E., Moreira V., Nascimento N.G., Lomonte B., Gutierrez J.M., Lopes de Melo R., Teixeira C. (2013). A Lys49 phospholipase A_2_, isolated from *Bothrops asper* snake venom, induces lipid droplet formation in macrophages which depends on distinct signaling pathways and the C-terminal region. BioMed Res. Int..

[50] Leiguez E., Motta P., Maia Marques R., Lomonte B., Sampaio S.V., Teixeira C. (2020). A Representative GIIA Phospholipase A_2_ Activates Preadipocytes to Produce Inflammatory Mediators Implicated in Obesity Development. Biomolecules.

[51] Murakami M., Nakatani Y., Atsumi G.I., Inoue K., Kudo I. (2017). Regulatory Functions of Phospholipase A_2_. Crit. Rev. Immunol..

[52] Guijas C., Perez-Chacon G., Astudillo A.M., Rubio J.M., Gil-de-Gomez L., Balboa M.A., Balsinde J. (2012). Simultaneous activation of p38 and JNK by arachidonic acid stimulates the cytosolic phospholipase A_2_-dependent synthesis of lipid droplets in human monocytes. J. Lipid Res..

[53] Jarc E., Kump A., Malavasic P., Eichmann T.O., Zimmermann R., Petan T. (2018). Lipid droplets induced by secreted phospholipase A_2_ and unsaturated fatty acids protect breast cancer cells from nutrient and lipotoxic stress. Biochim. Biophys. Acta Mol. Cell Biol. Lipids.

[54] Onal G., Kutlu O., Gozuacik D., Dokmeci Emre S. (2017). Lipid Droplets in Health and Disease. Lipids Health Dis..

[55] Bosch M., Sanchez-Alvarez M., Fajardo A., Kapetanovic R., Steiner B., Dutra F., Moreira L., Lopez J.A., Campo R., Mari M. (2020). Mammalian lipid droplets are innate immune hubs integrating cell metabolism and host defense. Science.

[56] Karagiannis F., Masouleh S.K., Wunderling K., Surendar J., Schmitt V., Kazakov A., Michla M., Holzel M., Thiele C., Wilhelm C. (2020). Lipid-Droplet Formation Drives Pathogenic Group 2 Innate Lymphoid Cells in Airway Inflammation. Immunity.

[57] Marschallinger J., Iram T., Zardeneta M., Lee S.E., Lehallier B., Haney M.S., Pluvinage J.V., Mathur V., Hahn O., Morgens D.W. (2020). Lipid-droplet-accumulating microglia represent a dysfunctional and proinflammatory state in the aging brain. Nat. Neurosci..

[58] Tall A.R., Yvan-Charvet L. (2015). Cholesterol, inflammation and innate immunity. Nat. Rev. Immunol..

[59] Schaftenaar F., Frodermann V., Kuiper J., Lutgens E. (2016). Atherosclerosis: The interplay between lipids and immune cells. Curr. Opin. Lipidol..

[60] Giannotti K.C., Weinert S., Viana M.N., Leiguez E., Araujo T.L.S., Laurindo F.R.M., Lomonte B., Braun-Dullaeus R., Teixeira C. (2019). A Secreted Phospholipase A_2_ Induces Formation of Smooth Muscle Foam Cells Which Transdifferentiate to Macrophage-like State. Molecules.

[61] Leiguez E., Zuliani J.P., Cianciarullo A.M., Fernandes C.M., Gutierrez J.M., Teixeira C. (2011). A group IIA-secreted phospholipase A_2_ from snake venom induces lipid body formation in macrophages: The roles of intracellular phospholipases A_2_ and distinct signaling pathways. J. Leukoc. Biol..

[62] Leiguez E., Giannotti K.C., Moreira V., Matsubara M.H., Gutierrez J.M., Lomonte B., Rodriguez J.P., Balsinde J., Teixeira C. (2014). Critical role of TLR2 and MyD88 for functional response of macrophages to a group IIA-secreted phospholipase A_2_ from snake venom. PLoS ONE.

[63] Chistiakov D.A., Melnichenko A.A., Myasoedova V.A., Grechko A.V., Orekhov A.N. (2017). Mechanisms of foam cell formation in atherosclerosis. J. Mol. Med..

[64] Son S.H., Goo Y.H., Chang B.H., Paul A. (2012). Perilipin 2 (PLIN2)-deficiency does not increase cholesterol-induced toxicity in macrophages. PLoS ONE.

[65] Turkish A., Sturley S.L. (2007). Regulation of triglyceride metabolism. I. Eukaryotic neutral lipid synthesis: “Many ways to skin ACAT or a DGAT”. Am. J. Physiol. Gastrointest. Liver Physiol..

[66] Rodriguez J.P., Leiguez E., Guijas C., Lomonte B., Gutierrez J.M., Teixeira C., Balboa M.A., Balsinde J. (2020). A Lipidomic Perspective of the Action of Group IIA Secreted Phospholipase A_2_ on Human Monocytes: Lipid Droplet Biogenesis and Activation of Cytosolic Phospholipase A_2_α. Biomolecules.

[67] Paine M.J., Desmond H.P., Theakston R.D., Crampton J.M. (1992). Purification, cloning, and molecular characterization of a high molecular weight hemorrhagic metalloprotease, jararhagin, from Bothrops jararaca venom. Insights into the disintegrin gene family. J. Biol. Chem..

[68] Saklatvala J., Dean J., Clark A. (2003). Control of the expression of inflammatory response genes. Biochemical Society Symposium.

[69] Piperi C., Papavassiliou A.G. (2012). Molecular mechanisms regulating matrix metalloproteinases. Curr. Top. Med. Chem..

[70] Teixeira Cde F., Fernandes C.M., Zuliani J.P., Zamuner S.F. (2005). Inflammatory effects of snake venom metalloproteinases. Mem. Inst. Oswaldo Cruz.

[71] Sunitha K., Hemshekhar M., Thushara R.M., Santhosh M.S., Sundaram M.S., Kemparaju K., Girish K.S. (2015). Inflammation and oxidative stress in viper bite: An insight within and beyond. Toxicon.

[72] De Toni L.G., Menaldo D.L., Cintra A.C., Figueiredo M.J., de Souza A.R., Maximiano W.M., Jamur M.C., Souza G.E., Sampaio S.V. (2015). Inflammatory mediators involved in the paw edema and hyperalgesia induced by Batroxase, a metalloproteinase isolated from *Bothrops atrox* snake venom. Int. Immunopharmacol..

[73] Gutierrez J.M., Escalante T., Rucavado A., Herrera C., Fox J.W. (2016). A Comprehensive View of the Structural and Functional Alterations of Extracellular Matrix by Snake Venom Metalloproteinases (SVMPs): Novel Perspectives on the Pathophysiology of Envenoming. Toxins.

[74] McKay D.G., Moroz C., De Vries A., Csavossy I., Cruse V. (1970). The action of hemorrhagin and phospholipase derived from *Vipera palestinae* venom on the microcirculation. Lab. Investig. J. Tech. Methods Pathol..

[75] Ownby C.L., Bjarnason J., Tu A.T. (1978). Hemorrhagic toxins from rattlesnake (*Crotalus atrox*) venom. Pathogenesis of hemorrhage induced by three purified toxins. Am. J. Pathol..

[76] Zelanis A., Oliveira A.K., Prudova A., Huesgen P.F., Tashima A.K., Kizhakkedathu J., Overall C.M., Serrano S.M.T. (2019). Deep Profiling of the Cleavage Specificity and Human Substrates of Snake Venom Metalloprotease HF3 by Proteomic Identification of Cleavage Site Specificity (PICS) Using Proteome Derived Peptide Libraries and Terminal Amine Isotopic Labeling of Substrates (TAILS) N-Terminomics. J. Proteome Res..

[77] Waheed H., Moin S.F., Choudhary M.I. (2017). Snake Venom: From Deadly Toxins to Life-saving Therapeutics. Curr. Med. Chem..

[78] Fox J.W., Serrano S.M. (2008). Insights into and speculations about snake venom metalloproteinase (SVMP) synthesis, folding and disulfide bond formation and their contribution to venom complexity. FEBS J..

[79] Oliveira A.K., Paes Leme A.F., Asega A.F., Camargo A.C., Fox J.W., Serrano S.M. (2010). New insights into the structural elements involved in the skin haemorrhage induced by snake venom metalloproteinases. Thromb. Haemost..

[80] Escalante T., Rucavado A., Fox J.W., Gutierrez J.M. (2011). Key events in microvascular damage induced by snake venom hemorrhagic metalloproteinases. J. Proteom..

[81] Castro A.C., Escalante T., Rucavado A., Gutierrez J.M. (2021). Basement membrane degradation and inflammation play a role in the pulmonary hemorrhage induced by a P-III snake venom metalloproteinase. Toxicon.

[82] Muniz J.R., Ambrosio A.L., Selistre-de-Araujo H.S., Cominetti M.R., Moura-da-Silva A.M., Oliva G., Garratt R.C., Souza D.H. (2008). The three-dimensional structure of bothropasin, the main hemorrhagic factor from *Bothrops jararaca* venom: Insights for a new classification of snake venom metalloprotease subgroups. Toxicon.

[83] Sanchez E.F., Schneider F.S., Yarleque A., Borges M.H., Richardson M., Figueiredo S.G., Evangelista K.S., Eble J.A. (2010). The novel metalloproteinase atroxlysin-I from Peruvian *Bothrops atrox* (Jergon) snake venom acts both on blood vessel ECM and platelets. Arch. Biochem. Biophys..

[84] Hong S.Y., Lee H., You W.K., Chung K.H., Kim D.S., Song K. (2003). The snake venom disintegrin salmosin induces apoptosis by disassembly of focal adhesions in bovine capillary endothelial cells. Biochem. Biophys. Res. Commun..

[85] Resiere D., Mehdaoui H., Neviere R. (2022). Inflammation and Oxidative Stress in Snakebite Envenomation: A Brief Descriptive Review and Clinical Implications. Toxins.

[86] Ferreira B.A., Deconte S.R., de Moura F.B.R., Tomiosso T.C., Clissa P.B., Andrade S.P., Araujo F.A. (2018). Inflammation, angiogenesis and fibrogenesis are differentially modulated by distinct domains of the snake venom metalloproteinase jararhagin. Int. J. Biol. Macromol..

[87] Gallagher P., Bao Y., Serrano S.M., Laing G.D., Theakston R.D., Gutierrez J.M., Escalante T., Zigrino P., Moura-da-Silva A.M., Nischt R. (2005). Role of the snake venom toxin jararhagin in proinflammatory pathogenesis: In vitro and in vivo gene expression analysis of the effects of the toxin. Arch. Biochem. Biophys..

[88] Ferraz C.R., Carvalho T.T., Fattori V., Saraiva-Santos T., Pinho-Ribeiro F.A., Borghi S.M., Manchope M.F., Zaninelli T.H., Cunha T.M., Casagrande R. (2021). Jararhagin, a snake venom metalloproteinase, induces mechanical hyperalgesia in mice with the neuroinflammatory contribution of spinal cord microglia and astrocytes. Int. J. Biol. Macromol..

[89] Moura-da-Silva A.M., Laing G.D., Paine M.J., Dennison J.M., Politi V., Crampton J.M., Theakston R.D. (1996). Processing of pro-tumor necrosis factor-alpha by venom metalloproteinases: A hypothesis explaining local tissue damage following snake bite. Eur. J. Immunol..

[90] Rucavado A., Escalante T., Teixeira C.F., Fernandes C.M., Diaz C., Gutierrez J.M. (2002). Increments in cytokines and matrix metalloproteinases in skeletal muscle after injection of tissue-damaging toxins from the venom of the snake *Bothrops asper*. Mediat. Inflamm..

[91] Fernandes C.M., Pereira Teixeira Cde F., Leite A.C., Gutierrez J.M., Rocha F.A. (2007). The snake venom metalloproteinase BaP1 induces joint hypernociception through TNF-alpha and PGE2-dependent mechanisms. Br. J. Pharmacol..

[92] Fernandes C.M., Zamuner S.R., Zuliani J.P., Rucavado A., Gutierrez J.M., Teixeira Cde F. (2006). Inflammatory effects of BaP1 a metalloproteinase isolated from *Bothrops asper* snake venom: Leukocyte recruitment and release of cytokines. Toxicon.

[93] Rucavado A., Lomonte B., Ovadia M., Gutierrez J.M. (1995). Local tissue damage induced by BaP1, a metalloproteinase isolated from *Bothrops asper* (Terciopelo) snake venom. Exp. Mol. Pathol..

[94] Clissa P.B., Laing G.D., Theakston R.D., Mota I., Taylor M.J., Moura-da-Silva A.M. (2001). The effect of jararhagin, a metalloproteinase from *Bothrops jararaca* venom, on pro-inflammatory cytokines released by murine peritoneal adherent cells. Toxicon Toxinology.

[95] Zigrino P., Kamiguti A.S., Eble J., Drescher C., Nischt R., Fox J.W., Mauch C. (2002). The reprolysin jararhagin, a snake venom metalloproteinase, functions as a fibrillar collagen agonist involved in fibroblast cell adhesion and signaling. J. Biol. Chem..

[96] Kamiguti A.S., Hay C.R., Theakston R.D., Zuzel M. (1996). Insights into the mechanism of haemorrhage caused by snake venom metalloproteinases. Toxicon.

[97] Coelho A.L., De Freitas M.S., Mariano-Oliveira A., Rapozo D.C., Pinto L.F., Niewiarowski S., Zingali R.B., Marcinkiewicz C., Barja-Fidalgo C. (2004). RGD- and MLD-disintegrins, jarastatin and EC3, activate integrin-mediated signaling modulating the human neutrophils chemotaxis, apoptosis and IL-8 gene expression. Exp. Cell Res..

[98] Rawlings N.D., Barrett A.J., Bateman A. (2012). MEROPS: The database of proteolytic enzymes, their substrates and inhibitors. Nucleic Acids Res..

[99] Braud S., Bon C., Wisner A. (2000). Snake venom proteins acting on hemostasis. Biochimie.

[100] Serrano S.M., Maroun R.C. (2005). Snake venom serine proteinases: Sequence homology vs. substrate specificity, a paradox to be solved. Toxicon.

[101] Megale A.A.A., Magnoli F.C., Guidolin F.R., Godoi K.S., Portaro F.C.V., Dias-da-Silva W. (2021). *Bitis arietans* Snake Venom and Kn-Ba, a Snake Venom Serine Protease, Induce the Production of Inflammatory Mediators in THP-1 Macrophages. Toxins.

[102] Menaldo D.L., Bernardes C.P., Pereira J.C., Silveira D.S., Mamede C.C., Stanziola L., Oliveira F., Pereira-Crott L.S., Faccioli L.H., Sampaio S.V. (2013). Effects of two serine proteases from Bothrops pirajai snake venom on the complement system and the inflammatory response. Int. Immunopharmacol..

[103] Mamede C.C., de Sousa B.B., Pereira D.F., Matias M.S., de Queiroz M.R., de Morais N.C., Vieira S.A., Stanziola L., de Oliveira F. (2016). Comparative analysis of local effects caused by *Bothrops alternatus* and *Bothrops moojeni* snake venoms: Enzymatic contributions and inflammatory modulations. Toxicon.

[104] Costa C.R.C., Belchor M.N., Rodrigues C.F.B., Toyama D.O., de Oliveira M.A., Novaes D.P., Toyama M.H. (2018). Edema Induced by a *Crotalus durissus* terrificus Venom Serine Protease (Cdtsp 2) Involves the PAR Pathway and PKC and PLC Activation. Int. J. Mol. Sci..

[105] Clemetson K.J. (2010). Snaclecs (snake C-type lectins) that inhibit or activate platelets by binding to receptors. Toxicon.

[106] Chang C.H., Chung C.H., Hsu C.C., Huang T.Y., Huang T.F. (2010). A novel mechanism of cytokine release in phagocytes induced by aggretin, a snake venom C-type lectin protein, through CLEC-2 ligation. J. Thromb. Haemost. JTH.

[107] Chung C.H., Wu W.B., Huang T.F. (2004). Aggretin, a snake venom-derived endothelial integrin alpha 2 beta 1 agonist, induces angiogenesis via expression of vascular endothelial growth factor. Blood.

[108] Wang W.J. (2008). Agglucetin, a tetrameric C-type lectin-like venom protein, regulates endothelial cell survival and promotes angiogenesis by activating integrin alphavbeta3 signaling. Biochem. Biophys. Res. Commun..

[109] Rego C.M.A., Francisco A.F., Boeno C.N., Paloschi M.V., Lopes J.A., Silva M.D.S., Santana H.M., Serrath S.N., Rodrigues J.E., Lemos C.T.L. (2022). Inflammasome NLRP3 activation induced by Convulxin, a C-type lectin-like isolated from *Crotalus durissus* terrificus snake venom. Sci. Rep..

[110] Sartim M.A., Riul T.B., Del Cistia-Andrade C., Stowell S.R., Arthur C.M., Sorgi C.A., Faccioli L.H., Cummings R.D., Dias-Baruffi M., Sampaio S.V. (2014). Galatrox is a C-type lectin in Bothrops atrox snake venom that selectively binds LacNAc-terminated glycans and can induce acute inflammation. Glycobiology.

[111] Castanheira L.E., Lopes D.S., Gimenes S.N.C., Deconte S.R., Ferreira B.A., Alves P.T., Filho L.R.G., Tomiosso T.C., Rodrigues R.S., Yoneyama K.A.G. (2017). Angiogenenic effects of BpLec, a C-type lectin isolated from *Bothrops pauloensis* snake venom. Int. J. Biol. Macromol..

[112] Yamazaki Y., Morita T. (2004). Structure and function of snake venom cysteine-rich secretory proteins. Toxicon Toxinol..

[113] Fry B.G., Vidal N., Norman J.A., Vonk F.J., Scheib H., Ramjan S.F., Kuruppu S., Fung K., Hedges S.B., Richardson M.K. (2006). Early evolution of the venom system in lizards and snakes. Nature.

[114] Yamazaki Y., Hyodo F., Morita T. (2003). Wide distribution of cysteine-rich secretory proteins in snake venoms: Isolation and cloning of novel snake venom cysteine-rich secretory proteins. Arch. Biochem. Biophys..

[115] Salazar E., Cirilo A., Reyes A., Barrientos M., Galan J., Sanchez E.E., Suntravat M. (2024). Snake venom cysteine-rich secretory protein from Mojave rattlesnake venom (Css-CRiSP) induces acute inflammatory responses on different experimental models. Toxicon X.

[116] Wang F., Li H., Liu M.N., Song H., Han H.M., Wang Q.L., Yin C.C., Zhou Y.C., Qi Z., Shu Y.Y. (2006). Structural and functional analysis of natrin, a venom protein that targets various ion channels. Biochem. Biophys. Res. Commun..

[117] Peichoto M.E., Mackessy S.P., Teibler P., Tavares F.L., Burckhardt P.L., Breno M.C., Acosta O., Santoro M.L. (2009). Purification and characterization of a cysteine-rich secretory protein from *Philodryas patagoniensis* snake venom. Comp. Biochem. Physiol. Toxicol. Pharmacol. CBP.

[118] Lodovicho M.E., Costa T.R., Bernardes C.P., Menaldo D.L., Zoccal K.F., Carone S.E., Rosa J.C., Pucca M.B., Cerni F.A., Arantes E.C. (2017). Investigating possible biological targets of Bj-CRP, the first cysteine-rich secretory protein (CRISP) isolated from *Bothrops jararaca* snake venom. Toxicol. Lett..

[119] Bernardes C.P., Menaldo D.L., Zoccal K.F., Boldrini-Franca J., Peigneur S., Arantes E.C., Rosa J.C., Faccioli L.H., Tytgat J., Sampaio S.V. (2019). First report on BaltCRP, a cysteine-rich secretory protein (CRISP) from *Bothrops alternatus* venom: Effects on potassium channels and inflammatory processes. Int. J. Biol. Macromol..

[120] Wang Y.L., Kuo J.H., Lee S.C., Liu J.S., Hsieh Y.C., Shih Y.T., Chen C.J., Chiu J.J., Wu W.G. (2010). Cobra CRISP functions as an inflammatory modulator via a novel Zn2+- and heparan sulfate-dependent transcriptional regulation of endothelial cell adhesion molecules. J. Biol. Chem..

[121] Deka A., Sharma M., Mukhopadhyay R., Devi A., Doley R. (2020). Naja kaouthia venom protein, Nk-CRISP, upregulates inflammatory gene expression in human macrophages. Int. J. Biol. Macromol..

[122] Izidoro L.F., Sobrinho J.C., Mendes M.M., Costa T.R., Grabner A.N., Rodrigues V.M., da Silva S.L., Zanchi F.B., Zuliani J.P., Fernandes C.F. (2014). Snake venom L-amino acid oxidases: Trends in pharmacology and biochemistry. BioMed Res. Int..

[123] Guo C., Liu S., Yao Y., Zhang Q., Sun M.Z. (2012). Past decade study of snake venom L-amino acid oxidase. Toxicon.

[124] Sahin M., Cingu A.K., Gozum N. (2013). Evaluation of cystoid macular edema using optical coherence tomography and fundus autofluorescence after uncomplicated phacoemulsification surgery. J. Ophthalmol..

[125] de Souza L.L., Stransky S., Guerra-Duarte C., Flor-Sa A., Schneider F.S., Kalapothakis E., Chavez-Olortegui C. (2015). Determination of Toxic Activities in *Bothrops* spp. Snake Venoms Using Animal-Free Approaches: Correlation Between In Vitro Versus In Vivo Assays. Toxicol. Sci. Off. J. Soc. Toxicol..

[126] Hiu J.J., Yap M.K.K. (2020). Cytotoxicity of snake venom enzymatic toxins: Phospholipase A_2_ and l-amino acid oxidase. Biochem. Soc. Trans..

[127] Costa T.R., Amstalden M.K., Ribeiro D.L., Menaldo D.L., Sartim M.A., Aissa A.F., Antunes L.M.G., Sampaio S.V. (2018). CR-LAAO causes genotoxic damage in HepG2 tumor cells by oxidative stress. Toxicology.

[128] Ali S.A., Stoeva S., Abbasi A., Alam J.M., Kayed R., Faigle M., Neumeister B., Voelter W. (2000). Isolation, structural, and functional characterization of an apoptosis-inducing L-amino acid oxidase from leaf-nosed viper (*Eristocophis macmahoni*) snake venom. Arch. Biochem. Biophys..

[129] Lazo F., Vivas-Ruiz D.E., Sandoval G.A., Rodriguez E.F., Kozlova E.E.G., Costal-Oliveira F., Chavez-Olortegui C., Severino R., Yarleque A., Sanchez E.F. (2017). Biochemical, biological and molecular characterization of an L-Amino acid oxidase (LAAO) purified from *Bothrops pictus* Peruvian snake venom. Toxicon.

[130] Wei J.F., Wei Q., Lu Q.M., Tai H., Jin Y., Wang W.Y., Xiong Y.L. (2003). Purification, characterization and biological activity of an L-amino acid oxidase from *Trimeresurus mucrosquamatus* venom. Sheng Wu Hua Xue Yu Sheng Wu Wu Li Xue Bao Acta Biochim. Biophys. Sin..

[131] Wei J.F., Yang H.W., Wei X.L., Qiao L.Y., Wang W.Y., He S.H. (2009). Purification, characterization and biological activities of the L-amino acid oxidase from *Bungarus fasciatus* snake venom. Toxicon.

[132] Corasolla Carregari V., Stuani Floriano R., Rodrigues-Simioni L., Winck F.V., Baldasso P.A., Ponce-Soto L.A., Marangoni S. (2013). Biochemical, pharmacological, and structural characterization of new basic PLA2 Bbil-TX from *Bothriopsis bilineata* snake venom. BioMed Res. Int..

[133] Micusan V.V., Borduas A.G. (1975). Papain hydrolysis of goat IgG immunoglobulins: A means of subclass characterization. Immunochemistry.

[134] Latinovic Z., Leonardi A., Koh C.Y., Kini R.M., Trampus Bakija A., Pungercar J., Krizaj I. (2020). The Procoagulant Snake Venom Serine Protease Potentially Having a Dual, Blood Coagulation Factor V and X-Activating Activity. Toxins.

[135] Megale A.A.A., Magnoli F.C., Kuniyoshi A.K., Iwai L.K., Tambourgi D.V., Portaro F.C.V., da Silva W.D. (2018). Kn-Ba: A novel serine protease isolated from *Bitis arietans* snake venom with fibrinogenolytic and kinin-releasing activities. J. Venom. Anim. Toxins Incl. Trop. Dis..

[136] Alomran N., Blundell P., Alsolaiss J., Crittenden E., Ainsworth S., Dawson C.A., Edge R.J., Hall S.R., Harrison R.A., Wilkinson M.C. (2022). Exploring the Utility of Recombinant Snake Venom Serine Protease Toxins as Immunogens for Generating Experimental Snakebite Antivenoms. Toxins.

[137] Boeno C.N., Paloschi M.V., Lopes J.A., Souza Silva M.D., Evangelista J.R., Dos Reis V.P., da S. (2022). Setúbal, S.; Soares, A.M.; Zuliani, J.P. Dynamics of action of a Lys-49 and an Asp-49 PLA_2_s on inflammasome NLRP3 activation in murine macrophages. Int. Immunopharmacol..

[138] Paloschi M.V., Boeno C.N., Lopes J.A., Rego C.M.A., Silva M.D.S., Santana H.M., Serrath S.N., Ikenohuchi Y.J., Farias B.J.C., Felipin K.P. (2022). Reactive oxygen species-dependent-NLRP3 inflammasome activation in human neutrophils induced by l-amino acid oxidase derived from *Calloselasma rhodostoma* venom. Life Sci..

[139] Silva M.D.S., Lopes J.A., Paloschi M.V., Boeno C.N., Rego C.M.A., de Oliveira Sousa O., Santana H.M., Dos Reis V.P., Serrath S.N., da S. (2022). Setúbal, S.; et al. NLRP3 inflammasome activation in human peripheral blood mononuclear cells induced by venoms secreted PLA_2_s. Int. J. Biol. Macromol..

[140] Ikenohuchi Y.J., Silva M.D.S., Rego C.M.A., Francisco A.F., da Silva Setubal S., Ferreira E.F.A.A., Boeno C.N., Santana H.M., Felipin K.P., de Lima A.M. (2023). A C-type lectin induces NLRP3 inflammasome activation via TLR4 interaction in human peripheral blood mononuclear cells. Cell. Mol. Life Sci. CMLS.

[141] de Zoete M.R., Palm N.W., Zhu S., Flavell R.A. (2014). Inflammasomes. Cold Spring Harb. Perspect. Biol..

[142] Kaiser E., Chiba P., Zaky K. (1990). Phospholipases in biology and medicine. Clin. Biochem..

[143] Kini R.M., Zhang C.Y., Tan B.K. (1997). Pharmacological activity of the interdomain segment between metalloproteinase and disintegrin domains. Toxicon.

[144] Chaves F., Leon G., Alvarado V.H., Gutierrez J.M. (1998). Pharmacological modulation of edema induced by Lys-49 and Asp-49 myotoxic phospholipases A_2_ isolated from the venom of the snake *Bothrops asper* (terciopelo). Toxicon.

[145] Zhang C., Medzihradszky K.F., Sanchez E.E., Basbaum A.I., Julius D. (2017). Lys49 myotoxin from the Brazilian lancehead pit viper elicits pain through regulated ATP release. Proc. Natl. Acad. Sci. USA.

[146] Lamkanfi M., Dixit V.M. (2012). Inflammasomes and their roles in health and disease. Annu. Rev. Cell Dev. Biol..

[147] Lamkanfi M., Dixit V.M. (2014). Mechanisms and functions of inflammasomes. Cell.

[148] Plato A., Willment J.A., Brown G.D. (2013). C-type lectin-like receptors of the dectin-1 cluster: Ligands and signaling pathways. Int. Rev. Immunol..

[149] Carvalho E., Oliveira W.F., Coelho L., Correia M.T.S. (2018). Lectins as mitosis stimulating factors: Briefly reviewed. Life Sci..

[150] Palm N.W., Medzhitov R. (2013). Role of the inflammasome in defense against venoms. Proc. Natl. Acad. Sci. USA.

[151] Nandana M.B., Bharatha M., Vishwanath B.S., Rajaiah R. (2024). Naja naja snake venom-induced local toxicities in mice is by inflammasome activation. Toxicon.

[152] Nandana M.B., Bharatha M., Praveen R., Nayaka S., Vishwanath B.S., Rajaiah R. (2024). Dimethyl ester of bilirubin ameliorates *Naja naja* snake venom-induced lung toxicity in mice via inhibiting NLRP3 inflammasome and MAPKs activation. Toxicon.

[153] Arbore G., Kemper C., Kolev M. (2017). Intracellular complement—The complosome—In immune cell regulation. Mol. Immunol..

[154] Ricklin D., Hajishengallis G., Yang K., Lambris J.D. (2010). Complement: A key system for immune surveillance and homeostasis. Nat. Immunol..

[155] Sarma J.V., Ward P.A. (2011). The complement system. Cell Tissue Res..

[156] Tambourgi D.V., van den Berg C.W. (2014). Animal venoms/toxins and the complement system. Mol. Immunol..

[157] Pidde-Queiroz G., Furtado Mde F., Filgueiras C.F., Pessoa L.A., Spadafora-Ferreira M., van den Berg C.W., Tambourgi D.V. (2010). Human complement activation and anaphylatoxins generation induced by snake venom toxins from *Bothrops genus*. Mol. Immunol..

[158] Pidde-Queiroz G., Magnoli F.C., Portaro F.C., Serrano S.M., Lopes A.S., Paes Leme A.F., van den Berg C.W., Tambourgi D.V. (2013). P-I snake venom metalloproteinase is able to activate the complement system by direct cleavage of central components of the cascade. PLoS Neglected Trop. Dis..

[159] Luchini L.S.G., Pidde G., Squaiella-Baptistao C.C., Tambourgi D.V. (2019). Complement System Inhibition Modulates the Pro-Inflammatory Effects of a Snake Venom Metalloproteinase. Front. Immunol..

[160] Ayres L.R., Recio Ados R., Burin S.M., Pereira J.C., Martins A.C., Sampaio S.V., de Castro F.A., Pereira-Crott L.S. (2015). Bothrops snake venoms and their isolated toxins, an L-amino acid oxidase and a serine protease, modulate human complement system pathways. J. Venom. Anim. Toxins Incl. Trop. Dis..

[161] Delafontaine M., Villas-Boas I.M., Pidde G., van den Berg C.W., Mathieu L., Blomet J., Tambourgi D.V. (2018). Venom from *Bothrops lanceolatus*, a Snake Species Native to Martinique, Potently Activates the Complement System. J. Immunol. Res..

[162] McIvor R.A., Lee-Pack L.R., Chan C.K. (1994). Occupational exposure and pulmonary function in health care workers in an aerosol pentamidine clinic. Chest.

[163] Yamamoto C., Tsuru D., Oda-Ueda N., Ohno M., Hattori S., Kim S.T. (2002). Flavoxobin, a serine protease from *Trimeresurus flavoviridis* (habu snake) venom, independently cleaves Arg726-Ser727 of human C3 and acts as a novel, heterologous C3 convertase. Immunology.

[164] Sun Q.Y., Bao J. (2010). Purification, cloning and characterization of a metalloproteinase from *Naja atra* venom. Toxicon.

[165] Wang R., Qiu P., Jiang W., Cai X., Ou Y., Su X., Cai J., Chen J., Yin W., Yan G. (2008). Recombinant fibrinogenase from *Agkistrodon acutus* venom protects against sepsis via direct degradation of fibrin and TNF-alpha. Biochem. Pharmacol..

[166] Lin X., Qi J.Z., Chen M.H., Qiu B.T., Huang Z.H., Qiu P.X., Chen J.S., Yan G.M. (2013). A novel recombinant fibrinogenase of Agkistrodon acutus venom protects against hyperacute rejection via degradation of complements. Biochem. Pharmacol..

[167] Tanaka G.D., Pidde-Queiroz G., de Fatima D.F.M., van den Berg C., Tambourgi D.V. (2012). Micrurus snake venoms activate human complement system and generate anaphylatoxins. BMC Immunol..

[168] Schaefer L. (2014). Complexity of danger: The diverse nature of damage-associated molecular patterns. J. Biol. Chem..

[169] Alam M.I., Quasimi H., Kumar A., Alam A., Bhagat S., Alam M.S., Khan G.A., Dhulap A., Ahmad Ansari M. (2022). Protective effects of novel diazepinone derivatives in snake venom induced sterile inflammation in experimental animals. Eur. J. Pharmacol..

[170] Chakrabartty S., Alam M.I., Bhagat S., Alam A., Dhyani N., Khan G.A., Alam M.S. (2019). Inhibition of snake venom induced sterile inflammation and PLA2 activity by Titanium dioxide Nanoparticles in experimental animals. Sci. Rep..

[171] Menon J.C., Nair B., Pati S., Pillay V.V., Mahapatra A., Sreekrishnan T.P., Vanuopadath M., John D., Nair S.B., Sahoo P.K. (2024). From neglect to equity in snakebite envenoming; what the ICMR-Collaborative Centre of Excellence (CCoE) targets. PLoS Neglected Trop. Dis..

[172] Ratanabanangkoon K. (2023). Polyvalent Snake Antivenoms: Production Strategy and Their Therapeutic Benefits. Toxins.

[173] Gutierrez J.M., Vargas M., Segura A., Herrera M., Villalta M., Solano G., Sanchez A., Herrera C., Leon G. (2020). In Vitro Tests for Assessing the Neutralizing Ability of Snake Antivenoms: Toward the 3Rs Principles. Front. Immunol..

[174] Bhatia S., Blotra A., Vasudevan K. (2022). Evaluating Antivenom Efficacy against *Echis carinatus* Venoms-Screening for In Vitro Alternatives. Toxins.

[175] Vanuopadath M., Rajan K., Alangode A., Nair S.S., Nair B.G. (2023). The Need for Next-Generation Antivenom for Snakebite Envenomation in India. Toxins.

[176] Thumtecho S., Burlet N.J., Ljungars A., Laustsen A.H. (2023). Towards better antivenoms: Navigating the road to new types of snakebite envenoming therapies. J. Venom. Anim. Toxins Incl. Trop. Dis..

[177] Vanuopadath M., Raveendran D., Nair B.G., Nair S.S. (2022). Venomics and antivenomics of Indian spectacled cobra (*Naja naja*) from the Western Ghats. Acta Trop..

[178] Rajan K., Alangode A., Menon J.C., Raveendran D., Nair S.S., Reick M., Nair B.G., Vanuopadath M. (2024). Comparative functional characterization and in vitro immunological cross-reactivity studies on *Daboia russelii* and *Craspedocephalus malabaricus* venom. Trans. R. Soc. Trop. Med. Hyg..

[179] Vanuopadath M., Shaji S.K., Raveendran D., Nair B.G., Nair S.S. (2020). Delineating the venom toxin arsenal of Malabar pit viper (*Trimeresurus malabaricus*) from the Western Ghats of India and evaluating its immunological cross-reactivity and in vitro cytotoxicity. Int. J. Biol. Macromol..

[180] Vanuopadath M., Sajeev N., Murali A.R., Sudish N., Kangosseri N., Sebastian I.R., Jain N.D., Pal A., Raveendran D., Nair B.G. (2018). Mass spectrometry-assisted venom profiling of *Hypnale hypnale* found in the Western Ghats of India incorporating de novo sequencing approaches. Int. J. Biol. Macromol..

[181] Jiang Y., Rex D.A.B., Schuster D., Neely B.A., Rosano G.L., Volkmar N., Momenzadeh A., Peters-Clarke T.M., Egbert S.B., Kreimer S. (2024). Comprehensive Overview of Bottom-Up Proteomics Using Mass Spectrometry. ACS Meas. Sci. Au.

[182] Gopalakrishnan M., Yadav P., Mathur R., Midha N., Garg M.K. (2021). Venom-Induced Consumption Coagulopathy Unresponsive to Antivenom After *Echis carinatus sochureki* Envenoming. Wilderness Environ. Med..

[183] Nuchpraryoon I., Garner P. (2000). Interventions for preventing reactions to snake antivenom. Cochrane Database Syst. Rev..

[184] Mahmood K., Naqvi I.H., Talib A., Salkeen S., Abbasi B., Akhter T., Iftikhar N., Ali A. (2010). Clinical course and outcome of snake envenomation at a hospital in Karachi. Singap. Med. J..

[185] de Silva H.A., Pathmeswaran A., Ranasinha C.D., Jayamanne S., Samarakoon S.B., Hittharage A., Kalupahana R., Ratnatilaka G.A., Uluwatthage W., Aronson J.K. (2011). Low-dose adrenaline, promethazine, and hydrocortisone in the prevention of acute adverse reactions to antivenom following snakebite: A randomised, double-blind, placebo-controlled trial. PLoS Med..

[186] Williams D.J., Jensen S.D., Nimorakiotakis B., Muller R., Winkel K.D. (2007). Antivenom use, premedication and early adverse reactions in the management of snake bites in rural Papua New Guinea. Toxicon.

[187] Gawarammana I.B., Kularatne S.A., Dissanayake W.P., Kumarasiri R.P., Senanayake N., Ariyasena H. (2004). Parallel infusion of hydrocortisone +/− chlorpheniramine bolus injection to prevent acute adverse reactions to antivenom for snakebites. Med. J. Aust..

[188] Russell J.J., Schoenbrunner A., Janis J.E. (2021). Snake Bite Management: A Scoping Review of the Literature. Plast. Reconstr. Surg. Glob. Open.

[189] Coppola M., Hogan D.E. (1994). When a snake bites. J. Am. Osteopath. Assoc..

[190] de Silva H.A., Ryan N.M., de Silva H.J. (2016). Adverse reactions to snake antivenom, and their prevention and treatment. Br. J. Clin. Pharmacol..

[191] Theakston R.D., Smith D.C. (1997). Antivenoms. BioDrugs Clin. Immunother. Biopharm. Gene Ther..

[192] Premawardhena A.P., de Silva C.E., Fonseka M.M., Gunatilake S.B., de Silva H.J. (1999). Low dose subcutaneous adrenaline to prevent acute adverse reactions to antivenom serum in people bitten by snakes: Randomised, placebo controlled trial. BMJ.

[193] Ariaratnam C.A., Sjostrom L., Raziek Z., Kularatne S.A., Arachchi R.W., Sheriff M.H., Theakston R.D., Warrell D.A. (2001). An open, randomized comparative trial of two antivenoms for the treatment of envenoming by Sri Lankan Russell’s viper (*Daboia russelii russelii*). Trans. R. Soc. Trop. Med. Hyg..

[194] Ontiveros S., Clark R.F., Minns A.B. (2021). Acute hypersensitivity reaction from administration of crotalidae immune F(ab’)2 antivenom. Clin. Toxicol..

[195] Malasit P., Warrell D.A., Chanthavanich P., Viravan C., Mongkolsapaya J., Singhthong B., Supich C. (1986). Prediction, prevention, and mechanism of early (anaphylactic) antivenom reactions in victims of snake bites. Br. Med. J. (Clin. Res. Ed.).

[196] Theakston R.D., Warrell D.A., Griffiths E. (2003). Report of a WHO workshop on the standardization and control of antivenoms. Toxicon.

[197] Lalloo D.G., Theakston R.D. (2003). Snake antivenoms. J. Toxicol. Clin. Toxicol..

[198] Leon G., Herrera M., Segura A., Villalta M., Vargas M., Gutierrez J.M. (2013). Pathogenic mechanisms underlying adverse reactions induced by intravenous administration of snake antivenoms. Toxicon.

[199] Ricciotti E., FitzGerald G.A. (2011). Prostaglandins and inflammation. Arterioscler. Thromb. Vasc. Biol..

[200] Shim J.S., Kang H., Cho Y., Shin H., Lee H. (2020). Adverse Reactions after Administration of Antivenom in Korea. Toxins.

[201] Deshpande R.P., Motghare V.M., Padwal S.L., Pore R.R., Bhamare C.G., Deshmukh V.S., Pise H.N. (2013). Adverse drug reaction profile of anti-snake venom in a rural tertiary care teaching hospital. J. Young Pharm. JYP.

[202] Morais V. (2018). Antivenom therapy: Efficacy of premedication for the prevention of adverse reactions. J. Venom. Anim. Toxins Incl. Trop. Dis..

[203] Hutchinson K.A. (2003). Granny sucks snakebite: A study of an envenomation. Aust. Crit. Care.

[204] Risch M., Georgieva D., von Bergen M., Jehmlich N., Genov N., Arni R.K., Betzel C. (2009). Snake venomics of the Siamese Russell’s viper (*Daboia russelli siamensis*)—Relation to pharmacological activities. J. Proteom..

[205] Laustsen A.H., Johansen K.H., Engmark M., Andersen M.R. (2017). Recombinant snakebite antivenoms: A cost-competitive solution to a neglected tropical disease?. PLoS Neglected Trop. Dis..

[206] Willard N.K., Salazar E., Oyervides F.A., Wiebe C.S., Ocheltree J.S., Cortez M., Perez R.P., Markowitz H., Iliuk A., Sanchez E.E. (2021). Proteomic Identification and Quantification of Snake Venom Biomarkers in Venom and Plasma Extracellular Vesicles. Toxins.

[207] Cavalcante J.S., Brito I., De Oliveira L.A., De Barros L.C., Almeida C., Rossini B.C., Sousa D.L., Alves R.S., Jorge R.J.B., Santos L.D.D. (2022). Experimental Bothropsatrox Envenomation: Blood Plasma Proteome Effects after Local Tissue Damage and Perspectives on Thromboinflammation. Toxins.

[208] Macedo J.K.A., Joseph J.K., Menon J., Escalante T., Rucavado A., Gutierrez J.M., Fox J.W. (2019). Proteomic Analysis of Human Blister Fluids Following Envenomation by Three Snake Species in India: Differential Markers for Venom Mechanisms of Action. Toxins.

[209] Rucavado A., Escalante T., Kalogeropoulos K., Camacho E., Gutierrez J.M., Fox J.W. (2020). Analysis of wound exudates reveals differences in the patterns of tissue damage and inflammation induced by the venoms of *Daboia russelii* and *Bothrops asper* in mice. Toxicon.

[210] Cavalcante J.S., Borges da Silva W.R.G., de Oliveira L.A., Brito I.M.C., Muller K.S., Vidal I.S.J., Dos Santos L.D., Jorge R.J.B., Almeida C., de Lima Bicho C. (2022). Blood plasma proteome alteration after local tissue damage induced by *Bothrops erythromelas* snake venom in mice. J. Proteom..

[211] Cavalcante J.D.S., de Almeida C.A.S., Clasen M.A., da Silva E.L., de Barros L.C., Marinho A.D., Rossini B.C., Marino C.L., Carvalho P.C., Jorge R.J.B. (2022). A fingerprint of plasma proteome alteration after local tissue damage induced by *Bothrops leucurus* snake venom in mice. J. Proteom..

[212] Sanchez-Castro E.E., Pajuelo-Reyes C., Tejedo R., Soria-Juan B., Tapia-Limonchi R., Andreu E., Hitos A.B., Martin F., Cahuana G.M., Guerra-Duarte C. (2020). Mesenchymal Stromal Cell-Based Therapies as Promising Treatments for Muscle Regeneration After Snakebite Envenoming. Front. Immunol..

[213] Hempel B.F., Damm M., Petras D., Kazandjian T.D., Szentiks C.A., Fritsch G., Nebrich G., Casewell N.R., Klein O., Sussmuth R.D. (2023). Spatial Venomics horizontal line Cobra Venom System Reveals Spatial Differentiation of Snake Toxins by Mass Spectrometry Imaging. J. Proteome Res..

[214] Buchberger A.R., DeLaney K., Johnson J., Li L. (2018). Mass Spectrometry Imaging: A Review of Emerging Advancements and Future Insights. Anal. Chem..

[215] Ghezellou P., Heiles S., Kadesch P., Ghassempour A., Spengler B. (2021). Venom Gland Mass Spectrometry Imaging of Saw-Scaled Viper, *Echis carinatus sochureki*, at High Lateral Resolution. J. Am. Soc. Mass Spectrom..

[216] Kazandjian T.D., Hamilton B.R., Robinson S.D., Hall S.R., Bartlett K.E., Rowley P., Wilkinson M.C., Casewell N.R., Undheim E.A.B. (2022). Physiological constraints dictate toxin spatial heterogeneity in snake venom glands. BMC Biol..

